# Exploring How Workflow Variations in Denaturation-Based Assays Impact Global Protein–Protein Interaction Predictions

**DOI:** 10.1016/j.mcpro.2025.101479

**Published:** 2025-12-11

**Authors:** Tavis J. Reed, Laura M. Haubold, Josiah E. Hutton, Olga G. Troyanskaya, Ileana M. Cristea

**Affiliations:** 1 Lewis-Sigler Institute for Integrative Genomics, Princeton University, Carl Icahn Laboratory, Princeton, New Jersey, USA; 2 Department of Computer Science, Princeton University, Princeton, New Jersey, USA; 3 Department of Molecular Biology, Princeton University, Princeton, New Jersey, USA; 4 Flatiron Institute, Simons Foundation, New York City, New York, USA

**Keywords:** protein–protein interactions, proteomics, interaction networks, thermal proximity coaggregation, ion-based proteome-integrated solubility alteration, timsTOF Ultra

## Abstract

Protein denaturation-based assays, such as thermal proximity coaggregation (TPCA) and ion-based proteome-integrated solubility alteration (I-PISA), are powerful tools for characterizing global protein–protein interaction (PPI) networks. These workflows utilize different denaturation methods to probe PPIs, *i.e.*, thermal- or ion-based. How denaturation differences influence PPI network mapping remained to be better understood. Here, we provide an experimental and computational characterization of the effect of the denaturation-based PPI assay on the observed PPI networks. We establish the value of both soluble and insoluble fractions in PPI prediction, determine the ability to minimize sample amount requirement, and assess different relative quantification methods during virus infection. Generating paired TPCA and I-PISA datasets, we define both overlapping sets of proteins and distinct PPI networks specifically captured by these methods. Assessing protein physical properties and subcellar localizations, we show that size, structural complexity, hydrophobicity, and localization influence PPI detection in a workflow-specific manner. We show that the insoluble fractions expand the detectable PPI landscape, underscoring their value in these workflows. Focusing on selected PPI networks (cytoskeletal and DNA repair), we observe the detection of distinct functional populations. Using influenza A infection as a model for cellular perturbation, we demonstrate that the integration of PPI predictions from soluble and insoluble workflows enhances the ability to build biologically informative and interconnected networks. Examining the effects of reducing starting material for TPCA assays, we find that PPI prediction quality remains robust when using a single well of a 96-well plate, a ∼500× reduction in sample input from usual workflows. Introducing simple workflow modifications, we show that label-free data-independent acquisition (DIA) TPCA yields performance comparable to the traditional tandem mass tag (TMT) data-dependent acquisition (DDA) TPCA workflow. This work provides insights into denaturation-based assays, highlights the value of insoluble fractions, and offers practical improvements for enhancing global PPI network mapping.

## Highlights

• Identify differences in physical properties of TPCA and I-PISA detected PPIs.
• Insoluble fractions in TPCA and I-PISA provide access to unique PPI populations.
• Optimize a DIA TPCA workflow for small sample amounts.
• Influenza A-driven PPI remodeling captured by soluble and insoluble TPCA workflows.

## Graphical Abstract

**Figure:**
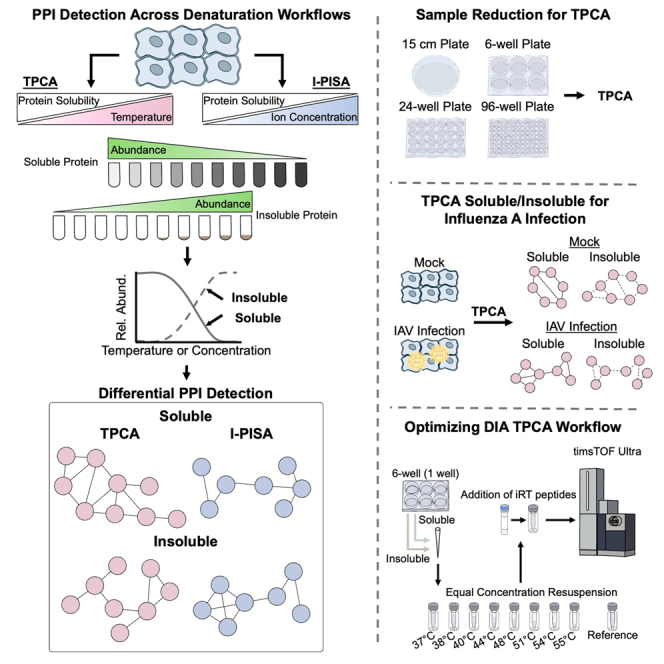

## In Brief

Here, we explore differences in denaturation-based assays for characterizing global protein–protein interaction (PPI) networks, assessing thermal proximity coaggregation (TPCA) and ion-based proteome-integrated solubility alteration (I-PISA) workflows. Specific physical properties of proteins are found to be influential for capturing divergent PPI networks. We establish that, apart from soluble fractions, the insoluble fractions obtained from these workflows access unique PPI populations. We optimize a soluble and insoluble TPCA workflow for low sample amount, applied to characterizing influenza A infection-driven PPI network remodeling.

Protein–protein interactions (PPIs) are essential for regulating cellular processes and responses to environmental cues during both health and disease states. These interactions collectively form dynamic, context-specific global PPI networks (1, 2, 3, 4). Accurately mapping these networks and characterizing their dynamics is crucial for advancing both fundamental biological knowledge and the understanding of how network disruptions are linked to the development of diseases, such as cancer and viral infections (5, 6, 7, 8, 9, 10, 11).

A variety of experimental methods have been developed for studying PPIs, including yeast two-hybrid screens (12, 13), protein microarrays (14), and mass spectrometry (MS)-based assays (15), such as affinity purification (16, 17), proximity labeling (18, 19, 20, 21, 22), crosslinking (23, 24), and co-fractionation (16, 25, 26). These efforts have facilitated the creation of large PPI repositories, such as BioGRID (27, 28), STRING (29), and BioPlex (1, 30, 31). These databases have significantly contributed to our understanding of protein functions, allowing us to build interconnected networks of PPIs towards a global view of protein associations and functions. Given that some of these repositories integrate data from heterogeneous biological conditions, they capture protein associations across conditions, while not necessarily representing a protein function or cellular response to a single biological context. Methods capable of globally mapping dynamic and context-specific PPI networks in a single experiment have been developed. Thermal proximity coaggregation (TPCA) (32, 33, 34, 35) and ion-based proteome-integrated solubility alteration (I-PISA) (36) are such MS-based methods. These methods utilize the principle that interacting proteins behave as a unit, becoming insoluble at similar rates when cells are subjected to denaturing conditions. In TPCA, cells are denatured through exposure to a temperature gradient, while in I-PISA, a ZnCl_2_ concentration gradient is applied. The remaining soluble protein from each of the resulting fractions is analyzed by MS, resulting in unique denaturation curves for all observed proteins. These curves are interpreted into PPI network predictions using either Euclidean distance calculations (37, 38) or machine learning approaches, such as the Tapioca framework (39). These methods have been shown to enable the probing of tens of thousands of *in vivo* and *ex vivo* PPIs for thousands of proteins in a single experiment (37, 38, 39, 40, 41, 42). Recent technological developments, as well as additional workflow considerations, suggest that more complete PPI networks can be derived from these methods.

An underexplored aspect of these denaturation-based methods is the potential value of the insoluble fraction produced during denaturation, which is typically not analyzed. For proteins that exhibit nominal denaturation curves (*i.e.*, those whose relative abundances decrease as temperature or ion concentration increases), it is expected that insoluble curves would be the direct inverse of their soluble counterparts. Thus, similar PPIs may be predicted by soluble and insoluble fractions. However, in both TPCA and I-PISA experiments, some proteins exhibit irregular denaturation profiles, where their relative abundances increase as temperature or ion concentration rises. These proteins, typically membrane-bound or nucleotide-associated, may display divergent behaviors in the insoluble fraction, and it remains unclear how similar their soluble and insoluble PPI predictions would be.

Another consideration is the ability to implement different relative quantification approaches in the MS analyses within denaturation-based workflows. In both TPCA and I-PISA, a single sample is split into multiple fractions (typically five to 10) before being subjected to denaturation. These fractions are commonly labeled using tandem mass tags (TMT) and analyzed by data-dependent acquisition (DDA) MS, enabling multiplexed MS analysis and minimizing missing values across denaturation curves. Label-free data-independent acquisition (DIA) TPCA workflows have also been explored, with studies showing improved curve resolution at the cost of a higher incidence of missing values, reducing the number of complete denaturation curves available for downstream PPI prediction (43, 44). Whether or not the improved denaturation curve resolution improves downstream PPI prediction compared to TMT-DDA TPCA has not yet been assessed. Furthermore, recent advancements in sample preparation techniques and instrument sensitivity have significantly reduced the amount of starting material required for proteomic experiments, enabling the analysis of rare cell types and even single cells. However, the effect of reducing sample input on downstream PPI predictions from denaturation-based assays remains unexplored.

**Fig. 1:**
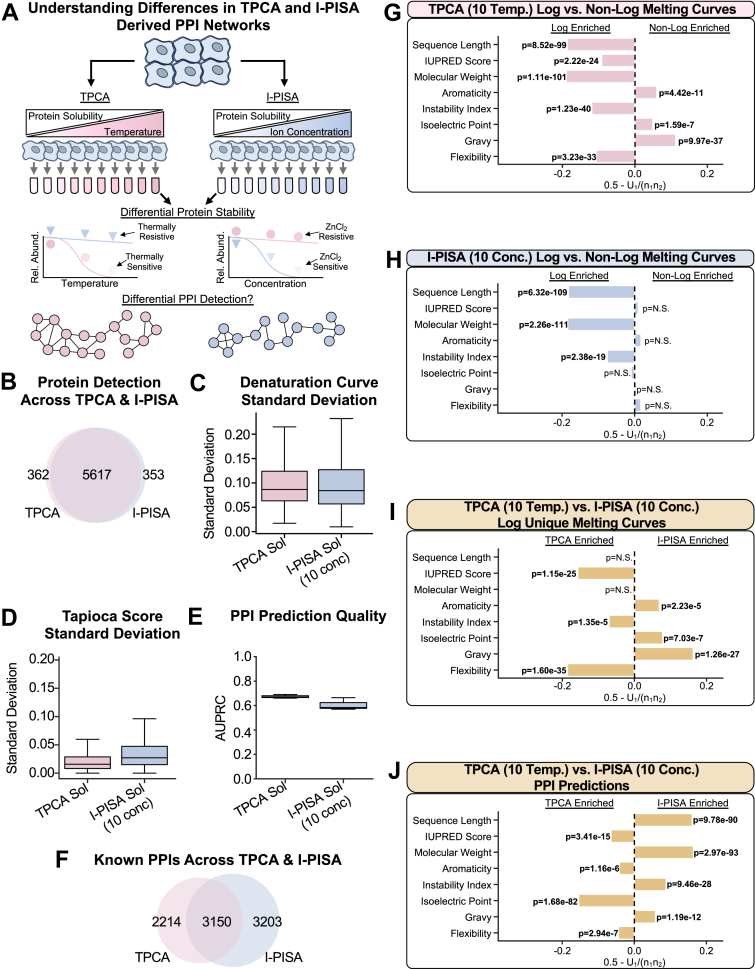
**Comparing PPI detection across TPCA and I-PISA experiments.***A*, schematic representation of the rationale for understanding the impact of the differential sensitivity of proteins to thermal- or ion-based denaturation on the detection of PPI networks. *B*, Venn diagram comparing the proteins detected across TPCA and I-PISA experiments. *C*, box plot comparing the standard deviation of denaturation curves between replicates for all proteins in a given experiment. The line within the box represents the median value and the whiskers represent the ±1.5 interquartile range. *D*, box plot comparing the standard deviation of Tapioca scores (39) assigned to a given pair of proteins between replicates for all pair or proteins in a given experiment. Box plot elements are the same as in *C*. *E*, box plot comparing PPI prediction quality, measured by area under a precision recall curve (AUPRC), across experimental conditions. Box plot elements are the same as in *C*. *F*, Venn diagram comparing known PPIs, with experimental evidence from STRING (29), BIOGRID (27, 28), or REACTOME (45), across TPCA and I-PISA experiments. *G–I*, bar plots showing the U-statistic derived values (X-axis) and associated multiple hypothesis test corrected *p*-values from the comparison of different amino acid sequence-predicted physical properties of proteins between proteins that display log-shaped and non-log-shaped curves for different workflows. *J*, bar plot showing the U-statistic derived values (X-axis) and associated multiple hypothesis test corrected *p*-value from the comparison of different sequence-predicted physical properties of known PPIs predicted as assembled from different workflows (see Methods). Across all panels, *p*-values were calculated using a two-sided Mann–Whitney *U* test, followed by multiple hypothesis test correction using the Bonferroni correction method, and for all experiments there are N = 3 biological replicates.

The distinct biochemical mechanisms used in TPCA and I-PISA workflows to denature proteins are also a consideration when aiming to maximize the depth and accuracy of the obtained global PPI networks (Fig. 1*A*). While the generated denaturation curve profiles have been compared, no direct comparison of the produced PPI networks has been performed to date. As noted by the developers of I-PISA, certain subsets of proteins exhibit resistance to denaturation in TPCA, I-PISA, or both workflows (36). It is likely that protein complexes exhibit differential sensitivity or resistance to thermal and ion-based denaturation, leading to divergent PPI predictions.

Here, we explore how differences in denaturation-based PPI assays affect the endpoint of observed PPI networks. We carefully assess different steps of these workflows, characterizing the similarities and differences of results obtained from TPCA and I-PISA, the value of insoluble fractions in PPI prediction, the ability to minimize sample amount requirements, and the use of different relative quantification methods. Specifically, we perform paired TPCA and I-PISA experiments in epithelial cells, collecting both soluble and insoluble fractions. We compare the resulting PPI networks both across (*i.e.*, soluble TPCA to soluble I-PISA) and within (*i.e.*, soluble to insoluble TPCA or I-PISA) denaturation methods. For soluble fractions, we demonstrate that TPCA and I-PISA produce substantial divergent PPI networks. Analysis of insoluble fractions highlights the value of these typically discarded samples for capturing different subsets of PPI networks. Our analyses point to specific physical properties of the proteins and the complexes they form as influential in the captured divergence of PPI networks, such as the isoelectric point for TPCA and intrinsically disordered regions for I-PISA. We further highlight the contribution of protein subcellular localization to the differential detection of PPIs across workflows. Applying soluble and insoluble TPCA workflows to studying influenza A infection, we demonstrate how the integration of PPIs detected across workflows can improve gained biological insight. Finally, using a timsTOF Ultra, we examine how decreasing sample input affects PPI prediction quality and coverage, additionally comparing PPI prediction quality between label-free DIA and TMT-DDA TPCA workflows. We show that, while reducing the amount of starting material decreases the number of proteins detected, the quality of PPI predictions remains consistent even when using ∼500× less starting material. Moreover, we demonstrate that with simple methodological improvements, label-free DIA TPCA is a suitable alternative to TMT-DDA TPCA for mapping PPI networks.

## Experimental Procedures

### Experimental Design and Statistical Rationale

For all experiments a sample size of three biological replicates was used. We consider a biological replicate the collection of a distinct biological cell sample. This number was chosen in order to be able to calculate statistical values for the results (*e.g.*, standard deviation and *p*-values). For the TMT TPCA and I-PISA experiments, the TPCA soluble workflow is considered the relevant control. For the low sample input label-free DIA TPCA experiments, the 15 cm data is considered the relevant control. For the influenza A TPCA experiment, the 0 h post infection (0 HPI, mock) is considered the relevant control. In the case of denaturation curves, curves are only discarded from any downstream analysis (excluding the effect of missing values on the number of curves analysis) if they contain any missing values. Apart from this, no other data points were excluded. There is a wide range of biologically relevant denaturation curve profiles, from the typical log-logistic profile to flat or increasing profiles (often observed for membrane proteins). Also noteworthy is the fact that the number of interactors a protein has can vary drastically (*e.g.*, a hub protein with hundreds of interactors vs. a protein within a smaller, defined complex), which can affect the ability to reliably detect outliers. Therefore, we do not remove or calculate outliers to preserve potentially biologically relevant signal. However, to control for noise in PPI predictions, we average scores across replicates. For calculating significance, the specific statistical test used to calculate *p*-values (either a two-sided Student’s *t* test, or a two-sided Mann-Whitney U test) is noted in the relevant figure caption and the test was implemented through Scipy (46) library. Where used, multiple hypothesis test correction (using the Bonferroni correction method) is noted in the figure caption and was implemented through the statsmodels (47) library. Data processing and large-scale analyses were performed using Python v.3.10.8, using the Python libraries SciPy (46), NumPy (48), Pandas (49, 50), Matplotlib (51), Seaborn (52), and Biopython (53). Cytoscape (54) was used to generate network visualizations. For boxplots, boxes show median, 25th and 75th percentile values, with the line within the box representing the median value, whiskers represent ± 1.5 interquartile range. Figures were created using Python and Microsoft PowerPoint.

### Cell lines, Virus Strains, Primary Culture, and Infections

A375 (ATCC CRL-1619) and A549 cells (ATCC CCL-185) were cultured in complete growth medium (DMEM supplemented with 10% fetal bovine serum [FBS]) supplemented with 1% penicillin-streptomycin at 37°C and 5% CO_2_. For influenza A infections, Influenza A H1N1 A/Puerto Rico/8/1934 (gift from Dr Thomas Shenk, Princeton University) was used. To improve virus yield in A549 cells, the influenza A stock was propagated 10 times in A549 cells prior to experiments, and virus stocks were stored at −80 °C until use. The virus was titered in A549 cells using immunofluorescent imaging using an Anti-Influenza A Virus Nucleoprotein antibody (abcam, ab128193) to detect infected cells. In the TPCA influenza A experiment, A549 cells were grown to 90 to 100% confluency. Next, the cells were infected at a multiplicity of infection (MOI) of 10 in a half culture volume of Opti-MEM (ThermoFisher, 11058021), supplemented with 0.25 μg/ml of TPCK trypsin (ThermoFisher, 20233) followed by incubation for 1 h. Following incubation, the infected media was removed, cells were washed with warm PBS, and then full volume complete growth medium was added. Infected cells were harvested at 18 h post infection (HPI), as detailed in the TPCA protocol below. As a control, uninfected cells were prepared using the same procedures as above, with the exception of virus infection; these cells were termed mock cells.

### TPCA and I-PISA Soluble and Insoluble MS sample Preparation

#### Collection and Denaturation

Cells were collected via trypsinization and then resuspended in 1× PBS, after which 50 μl of cells were aliquoted into PCR strips, five to 10 tubes for denaturation (depending on the experiment) and one additional tube for a non-denatured reference sample (*i.e.* another sample of cells from the same culture). Denaturation was then immediately performed on the relevant samples. Thermal denaturation for TPCA samples was performed using 5, 8, or 10 temperature points between 37 °C and 55 °C (5: 37 °C, 40.7 °C, 44.6 °C, 52.8 °C, 55.3 °C; 8: 37 °C, 38.2 °C, 40.3 °C, 43.9 °C, 48.1 °C, 51.2 °C, 53.5 °C, 55 °C; 10: 37 °C, 38.5 °C, 42 °C, 43.3 °C, 45 °C, 47 °C, 48.5 °C, 52 °C, 53.4 °C, 55 °C). Ion-based denaturation for the I-PISA samples was performed using five or 10 ZnCl_2_ concentrations between 0 μM and 800 μM (5: 0 μM, 150 μM, 250 μM, 350 μM, 550 μM; 10: 0 μM, 50 μM, 150 μM, 200 μM, 250 μM, 350 μM, 450 μM, 550 μM, 650 μM, 800 μM). After denaturation, 1.5× kinase buffer (75 mM HEPES pH 7.5, 15 mM MgCl_2_, 1.5× Halt protease and phosphatase inhibitor, 3 mM tris(2-carboxyethylphosphone) (TCEP)) were added to all samples, including reference samples. Samples were then snap-frozen and stored at −80 °C until preparation for MS analysis.

#### General MS Sample Preparation

Denatured samples were thawed on ice and then lysed in 1% Triton X-100, 0.1% Tween-20, and 0.5% sodium deoxycholate in 20 mM HEPES, 110 mM KOAc, 2 mM MgCl_2_, 1 μM ZnCl_2_, and 1 μM CaCl_2_ at pH 7.4 for 1 h. The samples were then further lysed via three freeze-thaw cycles. Reference samples were lysed in 5% SDS for 1 h. Denatured samples were then centrifuged at 20,000*g* for 20 min at 4 °C, pelleting the insoluble protein. The soluble proteins in the supernatant were then transferred to a new 1.5 ml low-bind tube. The remaining insoluble protein pellet was resuspended in 10% SDS. All samples (denatured soluble and insoluble, and reference) were then reduced and alkylated with 25 mM TCEP and 25 mM chloroacetamide at 55 °C for 30 min.

#### In-suspension Digestion

TMT TPCA, label free DIA 15 cm soluble and insoluble, 6-well soluble, influenza A, and all I-PISA experiments underwent in-suspension digestion. Reduced and alkylated samples were purified via methanol-chloroform precipitation. The resulting protein disks were then resuspended in 100 mM HEPES (pH 8.3). Following determination of sample protein concentration by BCA assay, samples were aliquoted into new low-bind tubes at a volume giving a concentration of 0.5 mg/ml for the 37 °C. The same volume used at 37 °C was used for all other temperatures within a given set. The aliquoted samples were then digested at 37 °C with 1 μg of sequencing grade trypsin (Thermo Fisher Scientific, catalog no. PI90059) for 16 h.

#### S-Trap Digestion

Label-free DIA 6-well insoluble, 24-well soluble and insoluble, and 96-well soluble and insoluble samples underwent purification and digestion using S-Trap Micro Spin Columns (Protifi). Phosphoric acid was added to samples to a final concentration of 1.2%, followed by the addition of 165 μl of 100 mM TEAB, pH 7.1 in 90% methanol and sample mixing by flicking. Samples were then placed on a S-Trap column and then centrifuged at 4000*g* until all volume had passed through the column. Then, five washes were performed in 165 μl of 100 mM TEAB, pH 7.1 in 90% methanol. Next, 100 μl of 50 mM TEAB, pH eight containing trypsin at a 1:50 ratio was added to the column. Digestion was performed for 16 h at 37 °C. Peptides were eluted with 40 μl of 25 mM TEAB followed by a second elution with 40 μl 0.2% aqueous formic acid. The elutions were combined, dried down by speed vacuum centrifugation, then resuspended in 50 μl water.

#### Sample Preparation for TMT-DDA MS Analysis

Digested samples were concentrated to near-dryness in a speed vacuum centrifuge before being resuspended in 16 μl of 5% ACN in water, followed by labeling by the addition of 95 μg of TMT reagent (TMT 11-plex, Thermo Fisher Scientific, catalog numbers 90406 and A34807). Samples were labeled for 1 h at room temperature while shaking at 1000 rpm. Samples were multiplexed, such that all temperature or ion concentrations from a single replicate of a single condition were in one plex. The exception was for the five-concentration I-PISA soluble and insoluble samples, which were multiplexed together by condition (*i.e.*, one plex contained one replicates of all I-PISA soluble and insoluble concentration points). Included in all the multiplexed samples was an additional reference sample, with aliquots from the exact same reference sample being used across all plexes. Labeling was stopped by the addition of 0.33% hydroxylamine (Sigma-Aldrich, catalog no. 467804–10 Ml) for 10 min. To evaluate the quality of labeling, a test mix was generated by combining 1 μl from each labeled sample within the multiplex with 89 μl of 1% trifluoroacetic acid (TFA, Thermo Fisher Scientific, catalog no. 28904). C18 stagetip desalting was performed on the test mix sample prior to being dried by speedvac then resuspended in 6 μl of 1% formic acid (FA) and 1% acetonitrile (ACN). This sample was then analyzed on a Q Exactive HF (QE-HF) mass spectrometer (MS) (Thermo Fisher Scientific). After confirming ≥98% labeling efficiency samples, a quantitative mixture was generated by combining 2 μl from each labeled sample in 300 μl of 0.1% TFA. A Pierce high pH reversed-phase peptide fractionation column (Thermo Fisher Scientific, catalog no. 84868) was then used to fractionate the mixture into eight fractions. These fractionated samples were then dried down by speedvac, then resuspended in 6 μl of 1% FA and 1% ACN prior to analysis on a QE-HF MS.

#### Sample Preparation for Label-Free DIA MS Analysis

TFA was added to digested samples to a final concentration of 1% TFA. Stage tips were prepared containing disks of SDB-RPS (Fisher Scientific). The stage tips were first washed with methanol, followed by a wash with buffer B (0.1% FA, 80% ACN), then two washes with buffer A (0.1% FA). Samples were then spun at 20,000*g* for 5 min to pellet any insoluble containments. Sample supernatant was then passed through stage tips, one sample per stage tip. Stage tips were then washed once with buffer A and twice with buffer B. Peptides were eluted using elution buffer (5% ammonium hydroxide, 80% ACN). Peptides were then dried down by speed vacuum centrifugation and then resuspended in 0.1% FA and 4% ACN, prior to analysis on a timsTOF Ultra (Bruker).

### Peptide Liquid Chromatography-tandem MS for TMT-DDA Samples

LC-MS/MS data acquisition and methods generation was performed using Xcalibur v.4 (Thermo Fisher Scientific). For all MS experiments, peptides were resolved for nanoscale liquid chromatography-MS analysis by a Dionex UltiMate 3000 nRSLC (Thermo Fisher Scientific) equipped with an EASY-Spray C18 column (2 μm particle size, 75 μm diameter, 500 m length; Thermo Fisher Scientific, catalog no. ES903), using a mixture of solvent A (0.1% FA, 99.9% LC-MS H_2_O) and solvent B (97% LC-MS ACN, 2.9% LC-MS H_2_O, 0.1% FA). For all DDA MS experiments a QE-HF instrument (Thermo Fisher Scientific) was used.

### Peptide Liquid Chromatography-Tandem MS for Label-free DIA Samples

LC-MS/MS analysis was performed with a nanoElute2 coupled to a timsTOF Ultra (Bruker) with 1 μl injections (150 ng of peptide on column for reference and 37 °C, less for higher temperature samples). The mobile phases were 0.1% LC-MS FA (Thermo Fisher Scientific) in 99.9% UHPLC-MS water (Fisher Scientific, buffer A) and 0.1% FA in 99.9% UHPLC-MS acetonitrile (Fisher Scientific, buffer B). For starting material exploration experiments, a two-column separation method was used. The trap column was a PepMap NEO (5 μm, 300 μm × 5 mm) C18 Trap column (Thermo Fisher Scientific) and the analytical column was a PepSep ULTRA (250 mm × 75 μm x 1.5 μm) C18 HPLC column (Bruker). For all other label-free DIA experiments a one-column separation method was used, using the same analytical column. All label-free DIA experiments used a 10 μm emitter (Bruker) attached to a CaptiveSpray Ultra source with a column toaster set to 50 °C. A linear 30-min gradient of 3% to 34% buffer B at a flow rate of 200 nl/min was used for peptide separation.

### TPCA TMT LC-MS/MS Analysis

Similar LC-MS/MS settings were used for both the test mix and quantitative mix samples, with differences noted below. MS/MS spectra were obtained in a full MS/data-dependent MS2 method, with full MS operated at 120,000 resolution, a maximum injection time (MIT) of 30 ms, target automatic gain control (AGC) of 3 × 10^6^, and a scan range of 350 to 1800 m/z. MS/MS scans were performed for the top 20 most intense precursor ions, with a 45,000 resolution, a 72 ms MIT, a target AGC of 1 × 10^5^, a normalized collision energy (NCE) of 34, and a fixed first mass of 100 m/z. Dynamic exclusion was used with a 25 s exclusion time.

#### Test Mix Samples

Peptides were resolved in a linear gradient of 6 to 18% solvent B over 60 min, then 18 to 29% solvent B over 30 min at a flow rate of 250 nl/min. An isolation window of 1.2 m/z was used.

#### Quantitative Mix Samples

Peptides were resolved on a linear gradient of 6 to 18% solvent B for 80 min followed by an 18 to 29% solvent B gradient for 30 min at a flow rate of 250 nl/min. An isolation window of 0.8 *m*/*z* was used.

### TPCA Label-free DIA LC-MS/MS analysis

For data-independent acquisition parallel accumulation serial fragmentation (dia-PASEF) analysis, the MS1 settings were set to start at 100 *m/z* and end at 1700 *m/z* in positive ion mode. For the TIMS settings, the mode was set to custom, with a starting 1/K_0_ of 0.65 V s/cm^2^ and an ending 1/K_0_ of 1.46 V s/cm^2^. Collision energy was linearly scaled to the ion mobility, ranging from 20 eV to 59 eV between 0.65 V s/cm^2^ and 1.46 V s/cm^2^. The ramp time was set to 50 ms with a 100% duty cycle and a ramp rate of 17.80 Hz. A 16 x 3 method was used for DIA windows, three groups of 16, spanning the mobility and m/z ranges. DDA was used to identify m/z and mobility ranges with the highest density of identifications in order to optimize window ranges using py_diAID (55). The estimated cycle time was 0.95 s.

### Peptide Identification and Quantification for TMT-DDA Samples

TMT MS/MS spectra were compared to protein sequences of a human reference proteome (downloaded 02/05/2024 from Bruker; 20,569 entries) and common contaminants using the SEQUEST algorithm in Proteome Discoverer v.2.5 (Thermo Fisher Scientific). A spectral recalibration node was used for the offline recalibration of mass accuracy. Only fully tryptic peptides with a maximum missed cleavage of two were included in the database. Cysteine carbamidomethylation (test and quant mix) and TMT labeling on peptide N termini and lysines (quant mix only) were included as static modifications. For dynamic modifications, the same TMT labeling was included for the test mix only. N-terminal methionine loss and acetylation, asparagine deamination, and methionine oxidation were included as dynamic modifications for both the test and quant mixes. A precursor mass tolerance of 4 ppm and fragment ion mass tolerance of 0.02 Da were used. Percolator (false discovery rate (FDR) of 1%) using a reversed sequence database search was used to calculate the FDR of matched spectra. The reporter quantifier node used an integration tolerance of 10 ppm, and the most confident centroid was used for the integration method. In the consensus workflow reporter ions quantifier node, a co-isolation threshold of 30 and an average reporter signal/noise threshold of eight was used.

### Peptide Identification and Quantification for Label-free DIA Samples

DIA data were analyzed with DIA-NN (version 1.8.2 beta 27), where a search was performed using a merged database of *Homo sapiens* appended with common contaminates (downloaded 02/05/2024 from Bruker, 20569 entries). A spectral library was produced with this database via DIA-NN, where the fragment ion *m/z* range was set to 200-1800 *m/z*, N-terminal methionine excision was enable, *in silico* digestion with trypsin with cleavage at lysine and arginine, a maximum of one missed cleavage, a peptide length between 7 and 30 amino acids, a precursor *m/z* range of 300 to 1,800, precursor charge state of one to 4, a fixed cysteine carbamidomethylation, with no dynamic modifications, and a spectral library was generated from the DIA runs. Precursor ion and fragment ion mass tolerance were both set to 15 ppm (as recommended by DIA-NN for the initial setting). For DIA-NN search settings, heuristic protein inference, MBR, use isotopologues, and unrelated runs were enabled. The neural network classifier was set to double-pass mode, the quantification strategy was set to QuantUMS (high precision), and cross-run normalization was set to off. Peptides and proteins filtered at a 1% FDR were used for downstream analysis.

### Processing of TMT-DDA TPCA and I-PISA Soluble and Insoluble Data

TMT-DDA TPCA and I-PISA data were processed as in our previous publications (39, 41), with a minor modification. This modification was the scaling of the raw values outputted by Proteome Discoverer v.2.4 using the derived ratios obtained from the test mix. These ratios were calculated from searching the RAW files in Proteome Discoverer v.2.4. and fitting the outputted ratios to a sigmoidal curve to correct for decreased protein solubility at higher temperatures and minimize technical variation between samples. After this scaling, median of median normalization using the reference channel was performed for within plex normalization. Briefly, the median value of all proteins within a single reference channel was calculated for each 11-plex. All proteins across all plexes were divided by the median of these median reference values. Next, cross-plex normalization was performed. For each individual protein the average reference value of that protein was calculated across all plexes. The resulting value as used to calculate a scaled reference value by dividing an individual protein’s reference channel value in a given plex by the previously calculated average. All values for that given protein were divided by the calculated scaled reference value. Finally, the data were fit to a three-parameter log-logistic equation:

(1) $$f\left(y\right)=c+\frac{1-c}{1+e^{b\left(\ln\left(y\right)-\ln\left(a\right)\right)}}$$

### Processing of Label-free DIA TPCA and I-PISA Soluble and Insoluble Data

First, using the pr_matrix and pg_matrix files outputted by DIA-NN, proteins with less than two identified proteins were discarded. Next, a per sample (one temperature of one condition of one replicate) scaling factor was computed by dividing the MS2.Signal value (found in the.stats file outputted by DIA-NN) associated with that sample by the mean MS2.Signal value across all analyzed samples. All abundance values with a sample were then multiplied by their respective sample-specific scaling factor. If iRT peptides were injected into the sample, then an iRT-abudance based sample-specific scaling factor was calculated and applied in the same manner. If a reference sample was included then for each protein, its reference abundance, specific to both condition and replicate, but not temperature, was divided by the median reference abundance for the given protein across all conditions and replicates. All abundance values for a given protein within a given condition and replicate were then multiplied by their respective condition and replicate specific reference scaling factor. For samples in which the protein was detected in the thermally denatured samples, but not the reference sample, all abundance values for a given protein within the given condition and replicate were multiplied by the median reference scaling factor respective to the given condition and replicate. Next, if the temperature samples (the samples that were thermally denatured) were scaled to be at equal protein concentration instead of equal volume within the experimental workflow, then all abundances for a given temperature were multiplied by that temperatures associated volume scaling factor (soluble volume scaling factors– 37 °C: 1.0, 38.2 °C: 1.0, 40.3 °C: 1.0, 43.9 °C: 1.0, 48.1 °C: 0.94, 51.2 °C: 0.73, 53.5 °C: 0.6, 55 °C: 0.5; insoluble volume scaling factors– 37 °C: 0.12, 38.2 °C: 0.13, 40.3 °C: 0.15, 43.9 °C: 0.22, 48.1 °C: 0.43, 51.2 °C: 0.67, 53.5 °C: 0.81, 55 °C: 1.0). Median of median normalization was performed next. First, the median abundance value across all proteins within each individual sample (one temperature of one condition of one replicate) was calculated. The median value of all of these median values was then calculated, and then all abundances were divided by this median value. Following this, for all proteins, per condition, the median abundance of a given protein across all temperatures and replicates was calculated. All abundance values for that protein within the specific condition were then divided by this median value. Next, proteins whose curves within a given condition and replicate contained a number of missing values above a given threshold were discarded (unless noted otherwise, this threshold was set to 0). Finally, the data were fit to the same three-parameter log-logistic equation as was used in the processing of TMT-DDA TPCA data.

### Calculating Denaturation Curve Standard Deviation

The standard deviation of denaturation curves was calculated by first assessing the standard deviation across replicates at each temperature or concentration point. The mean value of all these standard deviations was used as the standard deviation of the denaturation curve.

### Prediction of PPIs From TPCA Data

PPIs were predicted from TPCA data using Tapioca (39), an ensemble machine learning-based method that integrates dynamic MS data, such as from TPCA, with protein physical properties, protein domains, and tissue-specific functional networks. Here, a skin-specific functional network was used for all Tapioca predictions.

#### Evaluation of PPI Predictions

PPI prediction quality was assessed using the gold standard from Reed *et al*, 2024. This gold standard uses of a refined set of CORUM complex (56) as true positive examples. Negative examples were generated by taking all pairs of proteins which are not known to localize to the same subcellular compartments, per the Human Protein Atlas database (57), and for which no evidence interaction, experimental detected or computationally predicted, appears in the BioGRID (27, 28), REACTOME (45), MINT (58, 59), or STRING (29) databases.

#### Threshold for Calling a PPI Detected

A PPI was considered detected if it passed a dataset-specific Tapioca score cutoff. These cutoffs are 0.5 for TPCA soluble and insoluble (TMT), I-PSIA soluble (ten-concentrations), and eight-temperature DIA TPCA soluble and insoluble experiments, 0.6 for five-temperature DIA TPCA soluble and insoluble experiments, 0.7 for TPCA insoluble and I-PISA soluble and insoluble (five-concentrations), and 0.75 for influenza A TPCA. Cutoffs were chosen such that the false positivity rate across all experiments was roughly equal. Only for interactions involving a viral protein, a z-score cutoffs were used instead. For each viral protein, all of its PPI scores were converted into z-scores using only the distribution of the Tapioca scores associated with the given viral protein. A z-score cutoff of 3.5 was used for viral-viral PPIs and four was used for viral-host PPIs. This z-score approach was used due to the fact that no viral proteins are present in the tissue-specific functional networks used as an input for Tapioca. This shifts the score distribution for viral protein interactions to lower values, thus requiring an adjustment in calling confident viral PPIs.

#### Known PPIs

When known PPIs are discussed, these are PPIs with experimental evidence found in BioGRID (27, 28), STRING (29), or REACTOME (45).

#### CORUM Complexes

Briefly, complex scores were determined by calculating the average Tapioca score for all PPI pairs between subunits of the given complex. To be considered detected and assembled a CORUM complex had to both have a minimum of 50% of its subunits detected and have a score of greater than or equal to 0.4 for TPCA soluble and insoluble (TMT), I-PSIA soluble (ten-concentrations), and eight-temperature DIA TPCA soluble and insoluble experiments, 0.5 for five-temperature DIA TPCA soluble and insoluble experiments, 0.6 for TPCA insoluble and I-PISA soluble and insoluble (five-concentrations), and 0.75 for influenza A TPCA. Cutoffs were chosen such that the false positivity rate across all experiments was approximately equal.

#### Calculating Tapioca Score Standard Deviation

For calculating the standard deviation of Tapioca scores, we first assembled per replicate PPI scores into a single dictionary in which the given PPI was a key, and the value was a list (of up to three values for the three replicates) of all scores for the given PPI across all replicates. PPIs associated with a list of less than two values were discarded. We then calculated the standard deviation for each remaining PPI within the dictionary. It is these values that are plotted in boxplots in which Tapioca score standard deviation is discussed.

### Sequence Predicted Protein Physical Properties

#### Computing Protein Physical Properties

Protein physical properties were calculated from amino acid sequences using one of two methods. Protein molecular weight, aromaticity, instability index, isoelectric point, gravy, and flexibility were calculated using the SeqUtils.ProtParam module in BioPython (53). The IUPred3 webserver (60) was used to predict IUPred scores. Protein sequences were obtained from UniProt (61).

### Calculating PPI Overlap and Location Similarity

#### PPI Overlap

When calculating PPI overlap, only known PPIs that have passed the dataset-specific Tapioca score threshold are considered, except for the case of the BioPlex (31) related analysis, were all predicted PPIs and all BioPlex PPIs were considered. The overlap is calculated as the number of PPIs identified by both workflows, divided by the total number of PPIs detected by both workflows (PPIs detected in both are counted once, not twice). This is calculated on a per protein basis, in which the PPIs considered share the specific protein for which the overlap is being calculated (hence, calculated per individual protein interactome). In this calculation, the same PPI needs to be detected by both workflows to be considered as overlapping, and all known PPIs are considered.

#### Location Similarity

Subcellular localizations of proteins were obtained from the Human Protein Atlas (57). To calculate location similarity, a given proteins interactome (set of PPIs) was first filtered to include only known PPIs and PPIs with proteins for which subcellular localization information was available in the Human Protein Atlas in each workflow. Next, the total number of interactors that the given protein had in a given subcellular localization was calculated. Per subcellular localization, the difference in the number of interactions specific to the given localization detected by different workflows was calculated. This value was then divided by the total number of interactions identified within the given localization across both workflows being compared. One minus this value gives the location similarity value. The subcellular localizations used were nuclear plasma, nuclear membrane, nucleoli, actin filaments, cytosol, centriolar, endoplasmic reticulum, Golgi apparatus, intermediate filaments, microtubules, mitochondria, plasma membrane, and vesicles. In this calculation, it does not matter whether the exact same PPI was detected by both workflows, only the number of proteins within the given subcellular localization is considered.

### Analysis of Stable and Transient PPIs

#### Stable PPIs

Stable PPIs were obtained from the CORUM database (62), filtered to contain only complexes containing the following GO terms: D-glucose transmembrane transport, sodium ion transmembrane transport, calcium ion transmembrane transport, cell-cell adhesion, chloride transmembrane transport, potassium ion transmembrane transport, cilium assembly, intraciliary transport, mRNA splicing via spliceosome, norepinephrine transport, nuclear transport, nucleoside transmembrane transport, organic anion transport, oxygen transport, vesicle-mediated transport. All pairs of PPIs within a given complex, after filtering were then used as stable PPIs.

#### Transient PPIs

Transient PPIs were obtained from the SIGNOR database (63), which was first filtered to include only protein-protein interactions with Uniprot accessions for the pair of interacting proteins. The following SIGNOR-defined categories of mechanisms facilitating the PPI formation were accepted as transient PPIs: phosphorylation, dephosphorylation, polyubiquitination, ubiquitination, cleavage, delocalization, and acetylation. We defined an additional category of “other,” containing mechanisms with few representative proteins detected in our experiments, which contained the following SIGNOR defined categories: ADP-ribosylation, carboxylation, deacetylation, deglycosylation, demethylation, desumoylation, deubiquitination, glycosylation, guanine nucleotide exchange factor, hydroxylation, isomerization, lipidation, methylation, monoubiquitination, neddylation, oxidation, palmitoylation, post translational modification, s-nitrosylation, sumoylation, trimethylation, and tyrosination.

### Conditional Probabilities of Detecting a Protein in DIA Experiments

To better understand which temperature points were most responsible for missing values within a denaturation curve, we calculated the probability of detecting a protein at temperature A, given that the given protein was detected at temperature B. The results of these calculations are shown as asymmetric heatmaps.

### GO Term Enrichments

GO term enrichment was performed using HumanBase (64) (hb.flatironinstitute.org/). Given a set of genes or proteins, HumanBase detects functional modules using tissue-specific networks (in this study, a skin-specific network was used for A375 cells, and a lung-specific network for A549 cells). The GO term enrichment of these functional modules is then reported. The HumanBase output includes graphical representation of these modules/networks, wherein a node represents a protein, is colored by the module it belongs to, and its size represents its degree of connectivity within the network.

## Results

### Generating Sample-Paired TPCA and I-PISA Datasets

To understand the effect of the denaturation method on PPI network prediction, we generated sample-paired datasets using TPCA and I-PISA workflows. Briefly, epithelial cells were subjected to either a range of increasing temperatures or ZnCl_2_ concentrations. Using the soluble fractions derived from these denaturation steps, we compared the protein detection and downstream PPI prediction obtained from the TPCA or I-PISA workflows (Fig. 1*A* and Supplemental Table S1). Samples were labeled using tandem mass tags (TMT) for multiplexing within conditions and replicates (*i.e.*, the TPCA and I-PISA samples were each multiplexed as separate samples) and analyzed by data-dependent acquisition (DDA) MS on a QE HF instrument (Supplemental Fig. S1). Following the acquisition, these data were processed through our recently reported Tapioca framework (39) for PPI prediction. Tapioca is a machine learning-based method that integrates denaturation curves with protein physical properties, protein domains, and tissue-specific functional networks (39). A data normalization feature is included prior to PPI prediction (Supplemental Figs. S2 and S3; see Experimental Procedures). The Tapioca score, ranging from 0 to 1, represents the confidence with which the model predicts an interaction, with a value of one being the most confident. To make binary comparisons of PPIs across workflows, we used Tapioca score cutoffs that gave similar false positivity rates to account for variations in score distributions across workflows (Supplemental Fig. S4; see Experimental Procedures).

Comparing the total number and overlap of proteins identified by each workflow, we observed that TPCA and I-PISA performed similarly, each detecting nearly 6000 proteins, with an overlap of over 5600 (Fig. 1*B*). The differentially detected proteins were enriched (based on their gene ontology (GO) terms) in processes including DNA packaging and tRNA processing for TPCA, and organelle localization and translational elongation for I-PISA (Supplemental Fig. S5*A* and Supplemental Table S2). While the reproducibility of the denaturation curve profiles amongst the replicates of either TPCA or I-PISA workflows was comparable (Figs. 1*C* and Supplemental Fig. S5*B*), TPCA-based PPI predictions were slightly more consistent (Fig. 1*D*). Furthermore, TPCA slightly outperformed I-PISA in its PPI prediction quality (as measured by the area under a precision recall curve, AUPPRC, Fig. 1*E*). Of note, the TPCA workflow has undergone extensive optimization specifically to improve the capture of PPIs (38, 39), while the I-PISA workflow has been primarily implemented for solubility alteration assays rather than PPI prediction. Additionally, while the Tapioca framework has shown good performance on I-PISA data, it was originally trained on TPCA data and, thus, may have optimal performance for TPCA data. These considerations may explain why the performance of TPCA and I-PISA diverge when considering PPI-based metrics.

To determine whether these different workflows allow access to different subsets of PPI networks, we examined the correlation of Tapioca scores for individual proteins and their PPIs across workflows. Per protein, we observe a small, but positive, median Pearson correlation (0.192) between workflows (see Experimental Procedures and Supplemental Fig. S6*A*). Notably, there were significant outliers, with some proteins showing correlations near one and other near −1. To assess how these correlations impacted PPI predictions, we next filtered predicted PPIs to those ≥0.5 Tapioca scores (see Experimental Procedures). To facilitate the comparison of PPI prediction and diminish the contribution of prior specific method optimization, we focused on monitoring known PPIs, *i.e.*, those with experimental evidence present in STRING (29), BioGrid (27, 28), or REACTOME (45). We found an overlap of ∼3100 PPIs, while also observing many PPIs uniquely predicted by either TPCA (∼2200) or I-PISA (∼3200) (Fig. 1*F*). This capture of divergent PPI populations held true when assessing CORUM (62) complexes and all other (known and novel) predicted PPIs (Supplemental Fig. S6, *B* and *C*).

### Exploring the Role of Protein Physical Properties on denaturation Curves and PPI Predictions

Given that TPCA and I-PISA induce protein denaturation via distinct mechanisms, we examined whether differences in protein physical properties contribute to the observed differences in PPI networks. Physical properties were predicted for all detected proteins, analyzing protein sequence data using BioPython (53) and IUPred3 (60). The physical properties analyzed included protein sequence length, molecular weight, IUPred score (*i.e.*, a measure of structural disorder, where higher scores indicate greater disorder), aromaticity, instability index (*i.e.*, a measure of protein stability, where higher values indicate lower stability), isoelectric point, gravy (*i.e.*, a measure of hydrophobicity, where higher values indicate greater hydrophobicity), and flexibility.

Comparing the protein denaturation outcomes following the TPCA and I-PISA soluble workflows, we first asked what physical properties contribute to whether a protein is susceptible or not to either thermal or ZnCl2-based denaturation. For both TPCA and I-PISA, we found that properly denatured proteins (*i.e.*, exhibiting log-logistic curves) tended to have larger molecular weights and lower intrinsic stabilities (Fig. 1, *G* and *H*). However, specifically for TPCA, more flexible and disordered proteins had better susceptibility to thermal denaturation, while those with higher aromaticity, hydrophobicity, and isoelectric points tended to be more resistant. Next, we assessed the proteins that only denatured in either TPCA or I-PISA workflows (Fig. 1*I*). Thermal denaturation performed better on proteins that are more flexible, disordered, and less intrinsically stable. ZnCl_2_ denaturation performed better on proteins with high hydrophobicity, aromaticity, and isoelectric point. Hence, I-PISA may be suited for studying proteins with high stability, such as membrane-bound proteins.

We next asked whether the same physical properties influencing protein denaturation also contribute to the prediction of PPIs from TPCA and I-PISA data. Calculating the average physical properties of all pairwise PPIs predicted by each workflow, we compared the physical properties of uniquely detected known PPIs. Known PPIs captured by I-PISA were enriched for proteins with higher molecular weight and hydrophobicity, and lower intrinsic stability (Fig. 1*J*). Conversely, TPCA captured known PPIs between proteins that were more disordered and flexible and had higher aromaticity and isoelectric points. Interestingly, aromaticity and isoelectric point had reversed enrichment for PPI prediction when compared to protein susceptibility to denaturation. This suggests that the differences in PPI prediction go beyond the simple assessment of whether the given proteins are susceptible or not to denaturation. Rather, we hypothesize that, in addition to the susceptibility of a pair of proteins to denaturation, the specific intermolecular forces driving their interactions are also an important factor in enabling unique PPI detection across TPCA and I-PISA workflows.

## Insoluble TPCA and I-PISA Fractions Capture Unique PPI Populations

**Fig. 2:**
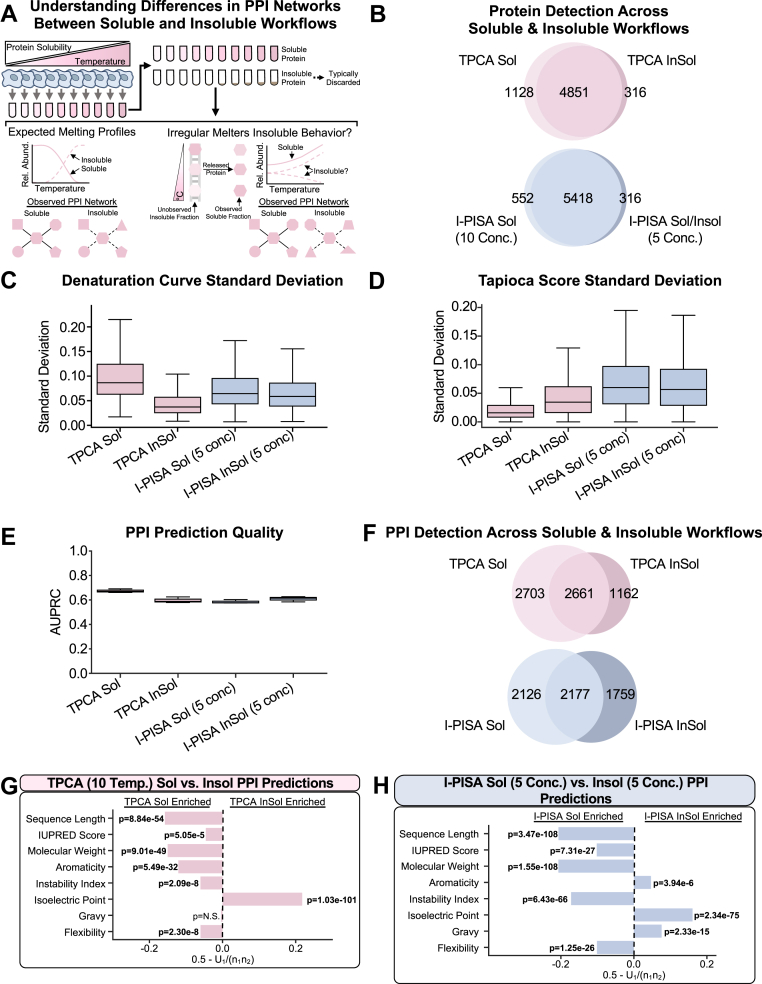
**Comparing PPI detection using soluble and insoluble fractions from TPCA and I-PISA workflows.***A*, schematic representation of the rationale for understanding the usefulness of insoluble fraction for PPI network prediction. *B*, Venn diagram comparing the proteins detected across soluble and insoluble TPCA and I-PISA experiments. *C*, box plot comparing the standard deviation of denaturation curves between replicates for all proteins in a given experiment. The line within the box represents the median value, and the whiskers represent the ±1.5 interquartile range. *D*, box plot comparing the standard deviation of Tapioca scores (39) assigned to a given pair of proteins between replicates for all pair or proteins in a given experiment. Box plot elements are the same as in *C*. *E*, box plot comparing PPI prediction quality, measured by area under a precision-recall curve (AUPRC), across experimental conditions. Box plot elements are the same as in *C*. *F*, Venn diagram comparing known PPIs, with experimental evidence from STRING (29), BIOGRID (27, 28), or REACTOME (45), across soluble and insoluble TPCA and I-PISA experiments. *G* and *H*, bar plot showing the U-statistic derived values (X-axis) and associated multiple hypothesis test corrected *p*-value from the comparison of different sequence-predicted physical properties of known PPIs found as assembled from different workflows (see Experimental procedures). Across all panels, *p*-values were calculated using a two-sided Mann-Whitney U test followed by multiple hypothesis test correction using the Bonferroni correction method. N = 3 biological replicates for all experiments.

While only the soluble fractions derived from denaturation steps within the TPCA and I-PISA workflows are traditionally analyzed by MS (34, 37, 39, 65), we next asked whether the insoluble fractions can expand the representation of proteins and subsets of PPIs (Fig. 2*A*). Hence, we produced sample-paired TPCA insoluble data for the 10 analyzed temperatures, comparing these data to the soluble counterparts (Supplemental Fig. S1). As expected, given the low abundance of many proteins in the insoluble fraction, we detected ∼800 fewer proteins compared to the TPCA soluble fractions (Fig. 2*B*). Interestingly, we observed improved denaturation curve reproducibility for the insoluble fractions (Fig. 2*C*, and Supplemental Fig. S5*B*). However, similar to the TPCA and I-PISA comparison, we again observed that the TPCA soluble data outperformed the insoluble data in terms of PPI prediction reproducibility and quality (Fig. 2, *D* and *E*). An important caveat to this is that, in addition to the prior methodological optimization of the TPCA soluble workflow, it is possible that there is a bias within the gold standard towards PPIs more readily detected in soluble fractions.

Given the drop in protein identifications between the soluble and insoluble TPCA data, we next asked whether multiplexing these fractions together may boost performance by minimizing missing values. Hence, we produced five concentration I-PISA soluble and insoluble samples, analyzed together as an 11-plex (with one reference channel). This workflow improved protein identification, detecting ∼550 proteins more than the TPCA insoluble data and only ∼200 proteins less than the 10 concentration I-PISA soluble data (Fig. 2*B*). Similar reproducibility in terms of both denaturation curves and PPI predictions was observed between the paired I-PISA soluble and insoluble data (Fig. 2, *C* and *D* and Supplemental Fig. S6*B*). However, the I-PISA insoluble data outperformed the soluble fractions in terms of PPI prediction quality (Fig. 2*E*). Consistent with our observations above, we found that TPCA and I-PISA soluble and insoluble workflows predicted many unique PPIs (Fig. 2*F*, and Supplemental Fig. S6, *B* and *C*).

Given the capture of distinct PPI populations, we considered whether protein physical properties contribute to this differential detection, akin to our observations for the soluble fractions. In the case of TPCA, the insoluble fraction better captured PPIs between proteins with high isoelectric points (Fig. 2*G*). For I-PISA, the insoluble-only PPIs were enriched for proteins with high aromaticity, hydrophobicity, and isoelectric point (Fig. 2*H*). Unlike the above soluble TPCA and I-PISA comparison, we find greater concordance between the physical properties that influence protein denaturation and unique PPI prediction in the insoluble fractions.

We next asked whether these observed correlations between physical properties and PPI predictions could be cross-validated within a set of context-specific PPIs. To enable this analysis, we utilized PPIs from the BioPlex Interactome (31), using both the 293T and HCT116 PPI sets. Firstly, prior to considering protein physical proteins, we noted a low overlap for individual protein interactomes between the TPCA/I-PISA datasets and the BioPlex (Supplemental Fig. S7*A*). This was expected (and in agreement with a previous report (39)) given the distinct modalities for capturing PPIs, which are, in this case, further exacerbated by the use of different cell types (A375 cells *vs.* 293T and HCT116 cells). Secondly, we assessed the physical properties for the proteins found in both the BioPlex and one of the TPCA or I-PISA datasets. This analysis confirmed our observations of distinct physical properties for PPIs found in the soluble and insoluble fractions. For example, TPCA insoluble PPIs also found in the BioPlex were enriched in proteins with higher isoelectric points and underrepresented in aromaticity (Supplemental Fig. S7*B*). In the case of the PPIs predicted from the soluble and insoluble I-PISA workflows, the comparison with the BioPlex confirmed the physical properties that we noted (Supplemental Fig. S7*C*). When focusing on the comparison between the soluble TPCA and I-PISA workflows (Supplemental Fig. S7*D*), the PPIs also captured in the BioPlex displayed many similar properties (sequence length, molecular weight, instability index, and isoelectric point) with what we noted before (Fig. 1). The IUPRED score, aromaticity, hydrophobicity, and flexibility were no longer significant (Supplemental Fig. S7*D*), likely due to the lower number of PPIs that overlapped with BioPlex.

Turning our attention to another important property of PPIs, we considered the PPI relative stability, testing the ability of the TPCA and I-PISA workflows to detect stable or transient PPIs (as assessed by Tapioca z-scores). We curated protein complexes from the CORUM and SIGNOR repositories to represent stable and transient interactions, respectively (see Experimental Procedures). The SIGNOR subset focused on PPIs involved in regulating post translational modifications (PTMs) (see Experimental Procedures). We found that all workflows predicted stable PPIs better than transient ones (Supplemental Fig. S7*E*). Subdividing transient interactions into PTM-driven types (*e.g.* acetylation, phosphorylation, ubiquitination, etc.), we observed some preferential detection. For example, interactions involving phosphorylation had statistically higher Tapioca z-scores than other transient interactions in the TPCA soluble workflow, as was the case for interactions involving acetylation in the I-PISA soluble workflow (Supplemental Fig. S7*F*). However, even with this subdivision, no transient PPIs showed predictions equivalent to those observed for stable PPIs across workflows. This is expected given the curve-driven modality of PPI prediction in these workflows, and hence the averaging of curves for proteins simultaneously existing in multiple transient states.

Overall, these results demonstrate how protein physical properties contribute to PPI characterization within TPCA and I-PISA workflows, as well as the ability of insoluble fractions to expand the depth and capture distinct PPI populations.

### Integration of Soluble and Insoluble Fractions Enhances the Coverage of PPI Networks

**Fig. 3:**
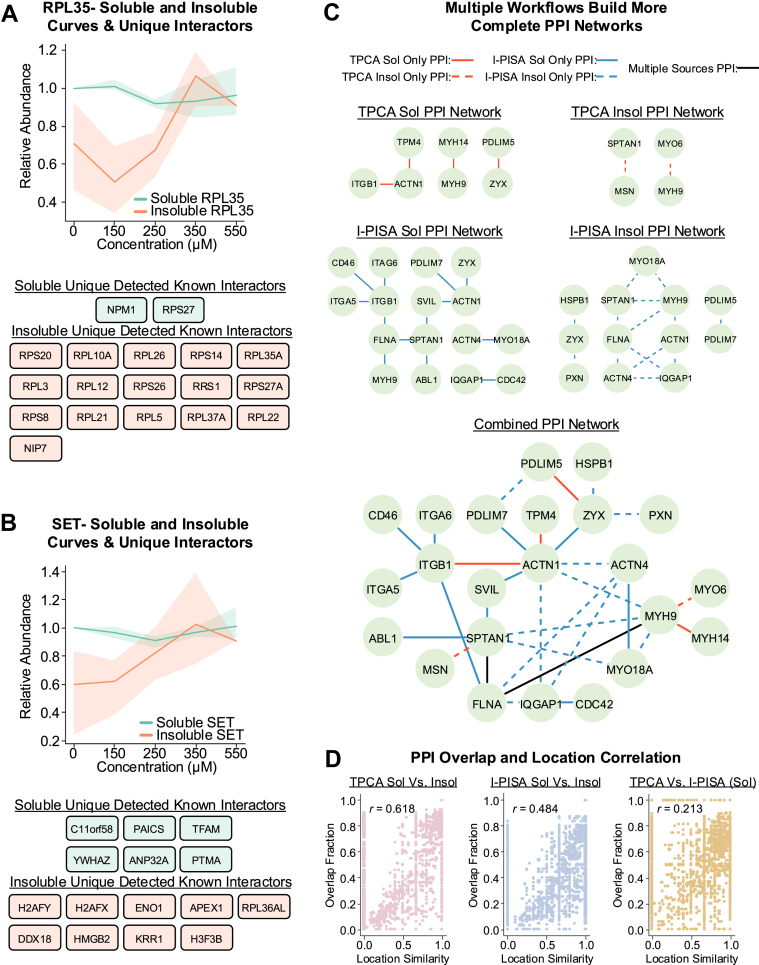
**Integrating PPI predictions using soluble and insoluble fractions across workflows improves PPI network coverage.***A* and *B*, soluble and insoluble I-PISA denaturation curve profiles for RPL35 (*A*) and SET (*B*) and their known interactors uniquely detected by soluble or insoluble workflows. In line plots, the solid line represents the median value and the shaded region represents the 95% confidence interval. *C*, a PPI network highlighting PPIs uniquely detected by different workflows. *D*, Scatter plot comparing the overlap fraction of known PPIs *versus* the location similarity of individual proteins across different experimental condition comparisons. Each dot represents a single protein with a single subcellular localization, and proteins with multiple subcellular localization have multiple dots. See the Experimental procedures section for how location similarity was calculated.

Given our finding that analyzing both the insoluble and soluble fractions may help to expand the interactomes for proteins that are more resistant to denaturation, we further investigated the coverage of selected protein interaction networks. As a first assessment, we considered proteins that displayed resistance to ion-based denaturation, such as RPL35 and SET. In the case of RPL35, its known interactions with other ribosomal subunit proteins were primarily detected by the I-PISA insoluble workflow (Fig. 3*A*). Thus, the I-PISA insoluble workflow better captures the presence of RPL35 within ribosomes. For SET, interactors uniquely detected by the I-PISA insoluble workflow were largely nuclear-localized proteins, known to bind DNA, while interactors uniquely detected in the soluble workflow were relatively enriched in cytoplasmic-localized proteins (Fig. 3*B*). These results suggest that, in addition to protein physical properties, several factors, such as protein subcellular localization, likely contribute to whether or not a given PPI is detected by a given workflow.

As a next assessment, we investigated proteins that, although being detected across workflows, the interactions captured were distinct between soluble and insoluble, as well as TPCA and I-PISA. An example is a network of cytoskeleton proteins that captured distinct functional populations for the myosin protein MYH9 (Fig. 3*C*). This protein had different interactions with other myosin proteins in each workflow, with these interactions likely having distinct biological functions and localizations. MYH9 is involved in regulating cell shape, cytokinesis, and intracellular tension (66), while MYO6, a reverse-direction motor protein (67), plays roles in endo- and exocytosis and homeostasis of the Golgi (68). Both MYH9 and MYO6 are known to interact with Na^+^/K^+^-ATPase α1 subunits and are believed to be involved in its tranport (69). The detection of the MYH9-MYO6 interaction may indicate that the population of MYH9 being probed by the TPCA insoluble workflow is enriched for the population of MYH9 involved in protein and vesicle transport. MYH14 shares similar functions and localizations to MYH9, but is less ubiquitously expressed across cell types (57, 70). The MYH14 interaction with MYH9 was reported to disrupt the role of MYH9 in GSK3β-mediated β-catenin ubiquitination and degradation (71). The detection of their interaction by the TPCA soluble analysis may imply that this workflow is capturing a population of MYH9 that is being more dominantly regulated by MYH14 than populations captured by the other workflows. MYO18A plays a role in linking the Golgi to the cytoskeleton, as well as in generating vesicles from the Golgi through tensile force (72, 73, 74). MYO18A can assemble with MYH9 and, given MYO18A’s lack of intrinsic motor ATPase function, it is thought that this interaction is important for the cytoskeletal roles of MYO18A (72). Given the capture of this interaction by the I-PISA insoluble assay, and the fact that this workflow was also the only one to identify the MYH9-ACTN1 interaction, this workflow may more prominently capture the population of MYH9 that is assembled with MYO18 A and interacting with cytoskeletal elements.

These differences in interaction functionality were also observed for other proteins within this network. Our results suggest that the TPCA soluble and I-PISA soluble and insoluble workflows may each be identifying different functional populations of ACTN1, involved in distinct cellular signaling complexes at the cell membrane. The TPCA soluble workflow identified ACTN1 interactions with TPM4, functioning to stabilize actin filaments (75, 76), and ITGB1, which contributes to sensing and responding to stimuli from the extracellular matrix through multiple signaling pathways (77, 78, 79). The I-PISA soluble workflow identified interactions between ACTN1 and PDLIM7, a scaffolding protein that links other proteins to actin filaments (80), ZYX, a mechanosensitive protein involved in cellular signal transduction and cytoskeletal remodeling (81, 82, 83), and SVIL, a protein that binds actin and membranes (84, 85). Finally, the I-PISA insoluble workflow identified ACTN1 interactions with MYH9, ACTN4, which shares high sequence homology with ACTN1 and is possibly involved in vesicle trafficking (86, 87, 88), and IQGAP1, a scaffolding protein with roles in cytoskeleton remodeling and cellular signaling as a regulator of the MAPK and Wnt/β-catenin signaling pathways (89, 90, 91).

Examining a network of DNA-binding proteins involved in DNA repair, replication, and sensing, we similarly found that the use of multiple workflows improves the coverage of known PPIs and likely captures distinct functional populations of proteins (Supplemental Fig. S8). For example, in this network, the formation of the MutS alpha complex (92), composed of MSH2 and MSH6, was uniquely captured by the I-PISA insoluble workflow. The I-PISA soluble workflow uniquely detected the interaction between MSH6 and MLH1, a component of the MutL alpha complex (93). The MutS alpha and MutL alpha complexes interact with one another to facilitate their functions in DNA repair (94), which is experimentally observed within the PPI network only through the use of both the soluble and insoluble I-PISA workflows.

Many proteins are functionally regulated, in part, by their recruitment to different subcellular locations, where they can engage in distinct PPI networks (11, 95, 96, 97, 98, 99, 100). Given that our analyses indicated that the soluble and insoluble workflows seem to preferentially identify interactors with different subcellular localizations, we next sought to determine whether this observation is more generalizable for PPI predictions across workflows (Fig. 3*D*). Using subcellular location annotations from the Human Protein Atlas (57, 101), we computed location similarity scores between the interactomes of the same protein within two different workflows for all proteins with subcellular localization information (see Experimental Procedures). A high location similarity score indicates that the interactomes compared consist predominantly of proteins localized to the same subcellular compartment, although these proteins need not be the exact same proteins. A low location similarity score indicates that workflows are probing different populations of a given protein that are in different subcellular locations. We then compared a protein's location similarity score to its interactome overlap fraction, in which a high score indicates interactions with the exact same proteins. This analysis showed a positive correlation between these values for both TPCA and I-PISA soluble versus insoluble comparisons. A smaller, but still positive, correlation was observed for TPCA versus I-PISA soluble workflows.

These findings highlight how different workflows can identify distinct functional and possibly differentially subcellularly localized, sets of PPIs for a given protein. By combining the PPI networks produced from each workflow, we can merge previously disconnected workflow-specific subgraphs into a unified graph. This approach provides a more comprehensive view of a proteins’ local PPI networks, while also, through leveraging network-based analyses, enhancing our understanding of how the interactions and behavior of distant proteins can influence a given protein.

### Irregularly Behaved Proteins are Distinct in TPCA and I-PISA Workflows

**Fig. 4:**
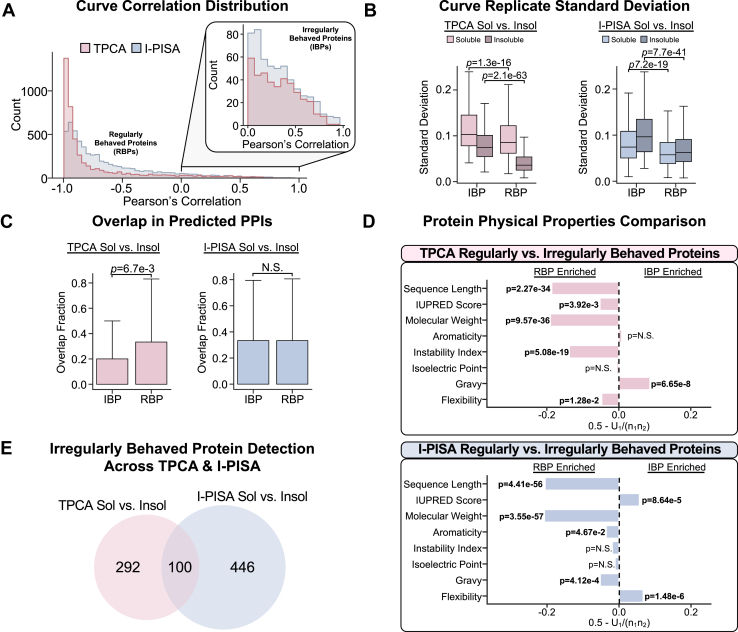
**Identifying irregularly behaved proteins.***A*, distribution of the Pearson’s correlation between the soluble and insoluble denaturation curves for each given protein. The red distribution compares TPCA soluble and insoluble. The blue distribution compares I-PISA soluble and insoluble. *B*, box plots comparing the standard deviation of Tapioca scores (39) assigned to a given pair of proteins between replicates for all pairs or proteins in an experiment. Box plots are split by experimental condition comparisons, and further subdivided by soluble versus insoluble, as well as by whether or not the protein’s soluble and insoluble denaturation curves are positively correlated. *p*-values are shown above the boxes. The line within the box represents the median value, and the whiskers represent the ±1.5 interquartile range. *C*, box plot comparing the fraction of PPIs detected across soluble and insoluble TPCA or I-PISA experiments. *p*-values are shown above the boxes. Box plot elements are the same as in *B*. *D*, bar plot showing the U-statistic derived values (X-axis) and associated multiple hypothesis test corrected *p*-value (using the Bonferroni correction method) from the comparison of different physical properties of proteins between regularly and irregularly behaved curves. E, Venn diagram comparing the irregularly behaved proteins in TPCA and I-PISA. Across all panels, *p*-values were calculated using a two-sided Mann–Whitney *U* test.

In our exploration and comparison of the data generated by each workflow, we identified a set of proteins whose paired soluble and insoluble denaturation curves exhibited unexpected relationships in both the TPCA and I-PISA workflows. While the vast majority of proteins displayed the expected strong negative correlation between their soluble and insoluble denaturation curves (regularly behaved proteins, RBP), a small subset of proteins, which we termed irregularly behaved proteins (IBPs), displayed positive correlations (Fig. 4*A* and Supplemental Fig. S9). In these cases, the relative abundance of the protein either increased (most commonly) or decreased in both the soluble and insoluble fractions as the temperature or ion concentration increased.

To better understand this irregular population, we first compared the standard deviations between replicates of IBP denaturation curves to those of RBPs. Both the soluble and insoluble denaturation curves of IBPs showed statistically significantly higher standard deviations than RBPs, albeit by a small margin, for both TPCA and I-PISA workflows (Fig. 4*B* and Supplemental Fig. S10). This suggests that IBP proteins may have a greater variability in response to denaturation. Next, we compared the overlap of known PPIs identified in both soluble and insoluble workflows, for either TPCA or I-PISA. Less overlap was observed for IBPs, compared to RBPs, in the TPCA workflow, which was not the case for I-PISA (Fig. 4*C*). Hence, the processes leading to IBPs in the TPCA workflow may influence which PPIs are observed between the soluble and insoluble workflows, while this is not observed for I-PISA. To understand sources for this distinction, we analyzed the physical properties of IBPs, compared to RBPs (Fig. 4*D*). For TPCA, we found that IBPs had higher hydrophobicity than RBPs, suggesting the representation of membrane-bound proteins. For I-PISA, IBPs had higher representation of disordered regions and were more flexible.

The significant differences in the physical properties of IBPs between the TPCA and I-PISA suggest that these proteins have different molecular functions and subcellular localizations. These findings further highlight how the integration of multiple workflows can help to uncover proteins with unusual behavior that may warrant more careful consideration in downstream PPI predictions. Additionally, the low overlap of IBPs between the TPCA and I-PISA workflows suggests that they can be minimized or avoided by selecting the appropriate workflow (Fig. 4*E*).

### The Effect of Starting Sample Abundance on PPI Predictions

**Fig. 5:**
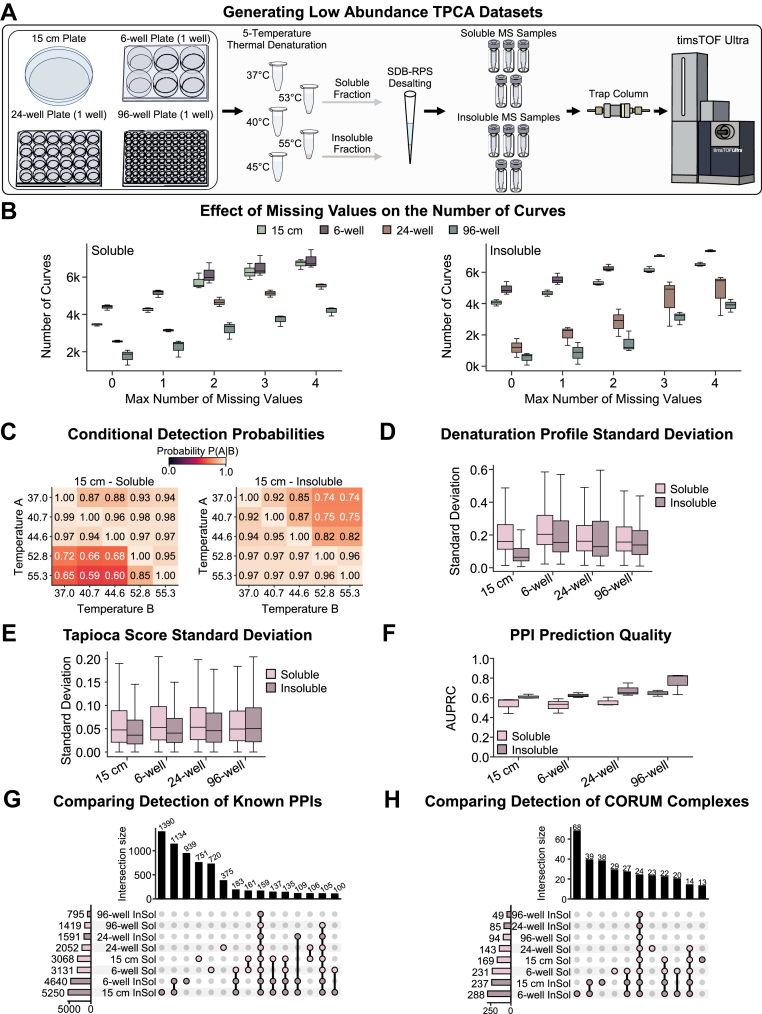
**Exploring low-sample amount DIA TPCA using a timsTOF Ultra.***A*, TPCA sample processing workflow for DIA analysis on a timsTOF Ultra (Bruker). *B*, box plot showing the number of curves by missing values for downstream PPI prediction at different thresholds of max number of missing values for any given curve. The line within the box represents the median value and the whiskers represent the ±1.5 interquartile range. *C*, the conditional probabilities of detecting a protein at temperature *A*, given the detection of the protein at temperature *B*. *D*, box plot comparing the standard deviation of denaturation curves between replicates for all proteins in an experiment. Box plot elements are the same as in *B*. *E*, box plot comparing the standard deviation of Tapioca scores assigned to a given pair of proteins between replicates for all protein pairs in an experiment. Box plot elements are the same as in *B*. *F*, box plot comparing PPI prediction quality, measured by area under a precision recall curve (AUPRC), across all experimental conditions. Box plot elements are the same as in *B*. *G*, upset plot comparing known PPIs, with experimental evidence from STRING (29), BIOGRID (27, 28), or REACTOME (45). *H*, upset plot comparing predicted assembled CORUM complexes (62). All experiments performed as N = 3 biological replicates.

Although advancements in mass spectrometry instrumentation and experimental and computational methods have enabled the analysis of low-input samples (102, 103, 104, 105, 106), the performance of denaturation-assays like TPCA when using reduced levels of starting material remained to be systematically tested. Specifically, it is not clear how this reduction in sample abundance might affect protein denaturation curves and the reliability of downstream PPI prediction. To address these questions, we prepared starting materials ranging from a confluent 15 cm plate of cells down to ∼500× less sample, obtained from a single confluent well of a 96-well plate (Fig. 5*A* and Supplemental Table S3). Based on our previous assessment of the number of temperature points needed for reliable PPI prediction (39), we generated five-temperature TPCA datasets for both soluble and insoluble fractions, which we analyzed using label-free DIA (data-independent acquisition) on a timsTOF Ultra (Bruker) instrument.

As expected, we found that decreasing the starting material reduced the number of detected peptides and proteins (Supplemental Fig. S11, *A* and *B*). For the soluble fractions, the decrease in protein identifications as the temperature increased (37 to 55 °C) changed from 29% for the 15 cm plate to 43% for the 96-well plate. For the insoluble fraction, as the temperature decreased (55 to 37 °C), the decrease in protein identifications increased from 23% for the 15 cm plate to 79% for the 96-well plate.

We next assessed the effect of missing values on protein identification and the number of curves (Fig. 5*B*). For the 15 cm plate data, 51% (soluble) and 63% (insoluble) of detected proteins had no missing values, with ∼63% (soluble) and 72% (insoluble) having only one missing value. By contrast, for the 96-well data, only 44% (soluble) and 17% (insoluble) of detected proteins had no missing values, while 57% (soluble) and 23% (insoluble) had only one missing value. These results suggest that the insoluble fraction is more adversely affected by decreasing starting material, implying that the insoluble workflow is less suited to low-input applications compared to the soluble fraction.

To identify the main sources of missing values, we calculated conditional probabilities for each temperature point (Fig. 5*C*, and Supplemental Fig. S11*C*). Not surprisingly, the temperatures with the lowest expected total protein abundance contributed the most to missing values. For soluble fractions, these temperatures were consistently 52.8 °C and 55.3 °C. For insoluble fractions, the largest number of missing values was noted for 37 °C and 40.7 °C for the 15 cm and 6-well samples, and for 44.6 °C when analyzing the 24- and 96-well samples.

Given that Tapioca provides optimal performance when using complete denaturation curves (no missing values), our subsequent analyses focused on proteins with no missing data. Examining these denaturation curves, we observed similar reproducibility (by standard deviation) across all input amounts, except for the insoluble 15 cm sample, which showed higher reproducibility than all other samples (Fig. 5*D* and Supplemental Fig. S12). Similarly, Tapioca scores showed consistent reproducibility across all starting material amounts (Fig. 5*E*). Furthermore, all input amounts exhibited comparable correlations in their Tapioca scores (Supplemental Fig. S13*A*). Most of the known PPIs identified in low-input samples were subsets of those identified in high-input samples, with missing PPIs in low-input samples likely due to incomplete denaturation curves.

Interestingly, we observed an apparent increase in PPI prediction quality as the amount of starting material decreased (Fig. 5*F*). This improvement may be partly attributed to an imbalance in the positive and negative examples available, as fewer curves with no missing values are retained as the starting material decreases. Specifically, negative examples were observed to decrease at a greater rate than positive examples, which were derived from well-studied and more abundant CORUM complexes. This change in balance may artificially inflate performance metrics. Nonetheless, evidence from Tapioca score correlations and the overlap of known PPIs indicates that lower-input samples provide predictions of similar quality to higher-input samples (Fig. 5, *G* and *H*, and Supplemental Fig. S13*B*).

Overall, while decreasing the starting material reduces the number of proteins for which PPIs can be predicted, it does not compromise the quality of the measured denaturation curves (with no missing values) or downstream PPI predictions. Furthermore, we observed across several performance metrics that using one well of a 6-well plate can provide similar data quality to that obtained from a 15 cm dish. This ability to use small sample amounts for TPCA analyses enables the definition of global PPI networks when working with limited sample contexts, such as patient-derived samples, profiling rare cell subpopulations, or many timepoints across a temporally dynamic biological context such as viral infection.

### Soluble and Insoluble TPCA Workflows Capture PPI Alterations upon Influenza A Infection

**Fig. 6:**
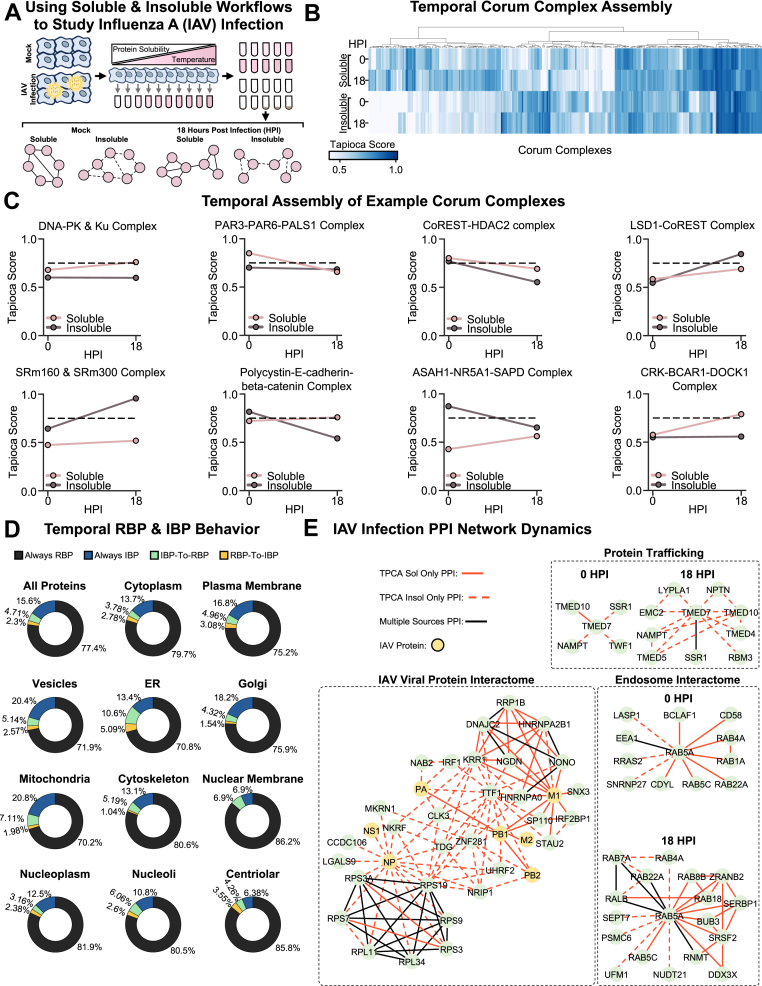
**Mapping PPI network dynamics upon influenza A infection using soluble and insoluble TPCA workflows.***A*, schematic representation of the influenza A experimental setup. *B*, clustermap of predicted CORUM complex assembly (by Tapioca score) across mock and infected conditions (HPI, hours post infection) and soluble and insoluble workflows. Each column represents a single CORUM complex; see Supplemental Table S6 for complex names and scores. *C*, line plots showing the temporal Tapioca scores of CORUM complexes across soluble and insoluble workflows. *D*, percentages of proteins, by subcellular localization, whose melting curves are regularly behaved (RBPs), irregularly behaved (IBPs), or that transition between these states across mock (0 HPI) and infected (18 HPI) states. *E*, temporal PPI networks centered on selected proteins (TMED7, RAB5A, influenza A viral proteins). N = 3 biological replicates for all experiments.

Given our finding that soluble and insoluble workflows provide different perspectives of PPIs within a basal cell state, we next asked whether these methods might facilitate characterizing changes in PPI networks upon a cellular perturbation. Using influenza A (IAV) infection as a model system, we implemented DIA TPCA soluble and insoluble workflows to monitor PPIs in lung epithelial (A549) cells. Uninfected (mock) cells were compared to 18 h post infection (HPI), and a reference sample was included at both time points to capture protein abundance changes (Fig. 6*A* and Supplemental Table S4).

At the proteome level, as expected, we observed increased relative abundances for immune factors at 18 HPI, including IFIT1, IFIT2, IFIT3, IRF9, ISG15, ISG20, OASL, and RIGI, indicating a host response to infection (Supplemental Fig. S14*A* and Supplemental Table S5). Next, we analyzed CORUM complexes, assessing their assembly or disassembly state based on comparison to gold standards (see Experimental Procedures). Many protein complexes displayed differential assembly states across mock and infected conditions, in both soluble and insoluble workflows (Fig. 6*B* and Supplemental Table S6). Some CORUM complexes, such as the DNA-PK-KU complex, involved in both DNA repair and immune sensing and signaling (41, 107), had increased predicted assembly confidence at 18 HPI, a finding only observed in the soluble data (Fig. 6*C*). Other complexes, like the CoREST-HDAC2 and LSD1-CoREST complexes, showed similar temporal trends of assembly across workflows, but with stronger signal in a given workflow (Fig. 6*C*). Specifically, the CoREST-HDAC2, involved in histone deacetylation (108), became disassembled during infection, while LSD1-CoREST, involved in histone demethylation (108), becomes assembled. This could represent an infection induced shift in chromatin regulation and gene expression. Finally, we observed that, for some complexes, such as the Polycystin-E-cadherin-beta-catenin complex involved in cell polarity and cell-cell adhesion (109), temporal assembly was observed from one workflow, while another workflow showed disassembly (Fig. 6*C*). This anticorrelated pattern of assembly could represent the translocation of these complexes in the cell or the probing of different subsets of these proteins. These results suggest that utilizing both soluble and insoluble workflows enhances the ability to characterize more complete PPI network dynamics within perturbed cellular states.

Using IAV infection as a perturbation model system, we assessed the dynamic nature of regularly or irregularly behaved proteins (RBPs and IBPs). When examining all detected proteins, we found that ∼7% of proteins transited between RBP and IBP states (∼5% IBP-to-RBP, ∼2% RBP-to-IBP) (Fig. 6*D*). Noteworthy, these transiting proteins seemed to be primed for such changes in curve behavior, as these proteins had soluble and insoluble curve correlations that were on average closer to zero compared to proteins that remained consistently as RBPs or IBPs (Supplemental Fig. S14*B*). We next considered the subcellular localizations of these transiting and stable proteins (Fig. 6*D*). Interestingly, specific subcellular compartments, such as the plasma membrane, Golgi, vesicles, and the mitochondria, had higher proportions of always IBPs (16.8%, 18.2%, 20.4%, 20.8%, respectively), suggesting that membranous organelles may be enriched for IBPs. The ER was prominently featured for proteins displaying IBPs-to-RBPs transitions (10.6%) during IAV infection. The ER is known to undergo extensive remodeling during IAV infection (110, 111, 112), and this transition in protein curve properties may be reflective of this remodeling.

To better understand how the infection-induced alterations in PPIs are differentially captured by distinct workflows, we selected representative interactomes. Proteins were selected based on a combined criteria of (1) having distinct PPIs captured by soluble and insoluble workflows, (2) transiting between IBP and RBP states across conditions, and (3) being relevant in biological processes regulated during IAV infection. TMED7, involved in protein trafficking and known to localize to the ER, Golgi, and vesicles (113, 114), gained many PPIs between the mock and infected states. Several of these gained interactions were with other TMED proteins, also involved in protein transport. An interaction was also gained with EMC2, involved in protein insertion at the ER (115). Interestingly, the only lost interaction was with TWF1, an actin-binding protein involved in endocytosis and endocytic organelle localization (116) (Fig. 6*E*). Together, this could represent dynamic protein transport at and around the ER during infection. In the case of RAB5A, involved in early endosome and plasma membrane fusion (117), PPI detection was more evenly spread between soluble and insoluble workflows (Fig. 6*E*). As with TMED7, we observed an overall increase in interactions during IAV infection, particularly with other RAB proteins. RAB proteins play important roles in IAV infection, with RAB5A functioning in the entry and early trafficking of IAV virions in endosomes (118, 119) and RAB11 being involved in viral ribonucleoprotein trafficking from the nucleus to the plasma membrane (112). These PPI alterations may reflect the infection-induced modulation of endosomes. As expected, ACTN1 had many interactions in both mock and IAV-infected states (Supplemental Fig. S14*C*). While there was a more balanced spread of PPIs detected across soluble and insoluble workflows in mock, the majority of interactions were only detected by the insoluble fraction during IAV infection. In both mock and infection conditions, the ACTN1 interactome was distinct to that observed in A375 cells (Fig. 3*C*). Many of the interactions detected in the mock condition were with other cytoskeletal proteins, including ACTN4, ZYX, CTTN, LASP1, VCL, and ACTR3 (Supplemental Fig. S14*C*). However, in mock, ACTN1 also had interactions with RTN4, involved ER tubule formation and stabilization (120), as well as TGOLN2, involved in Golgi-related membrane trafficking (121). Although many ACTN1 cytoskeletal interactions were preserved in infection, both RTN4 and TGOLN2 were lost (Supplemental Fig. S14*C*). The cytoskeleton is known to be remodeled during IAV infection (122), and the lack of detection for these interactions may represent remodeling of contacts between the ER and Golgi with the cytoskeleton.

We next explored the interconnected interactome of several influenza A viral proteins (Fig. 6*E*). In the soluble fraction, we observed the assembly of the viral polymerase complex (123), with PB1 interactions with PA and PB2. Also evident was the interaction between the viral M1 protein and STAU2, an RNA-binding host protein involved in microtubule-dependent transport of RNA (124) and known to play a role in influenza A H5N1 replication in chickens (125). In the insoluble fraction, we observed the interaction of the viral nucleoprotein (NP), reported to interact with ribosomal biogenesis factors (126), with several small and large ribosomal subunit proteins. In this fraction, we also observed an interaction between the NP and LGALS9, a galectin protein known to play a regulatory role in influenza A infection (127). We also observed many novel interactions, such as between the viral polymerase complex and TTF1, a host protein involved in replication fork arrest, regulation of RNA polymerase I transcription, and ribosomal gene transcription termination (128, 129).

Our findings demonstrate the utility of integrating soluble and insoluble TPCA workflows for gaining a more detailed picture of the remodeling of PPI networks during IAV infection. In addition to a better understanding of PPI assembly and disassembly, determining protein denaturation characteristics through soluble and insoluble melting curve correlation (RBPs vs IBPs) may enable insights into larger-scale modulations, such as organelle remodeling. This analysis highlights the potential benefits of utilizing multiple workflows, in this case, soluble and insoluble TPCA workflows, to improve the capture of PPI network alterations across dynamic biological states.

### Comparing Refined Label-free DIA TPCA Workflow to TMT DDA TPCA

Given our success with applying TPCA to limited sample amounts, in conjunction with DIA-based relative quantification, we next sought to better understand the effect of the type of relative MS quantification used within a TPCA workflow on the PPI prediction quality and network coverage. Recent studies have demonstrated the ability to convert the TPCA workflow from a TMT-DDA to a label-free DIA format (43, 130), and our results above also support this. Label-free DIA offers several advantages over TMT, including greater flexibility in selecting the number of temperature points for analysis, and reduced ratio compression and material costs. However, label-free DIA TPCA introduces a higher incidence of missing values compared to TMT-DDA TPCA (43, 44), which can critically limit the number of complete (zero missing values) denaturation curves available for downstream PPI predictions. This is also evident in our analyses, as the TMT-DDA TPCA soluble dataset (from a 15 cm plate) contained nearly 6000 complete denaturation curves (Fig. 1*B*), while the DIA TPCA soluble datasets provided ∼3500 and ∼4400 complete curves from an equivalent (15 cm plate) or the best performing (6-well) samples, respectively (Fig. 5*B*). This loss of complete denaturation curves limits the numbers of proteins that can be incorporated in PPI networks.

**Fig. 7:**
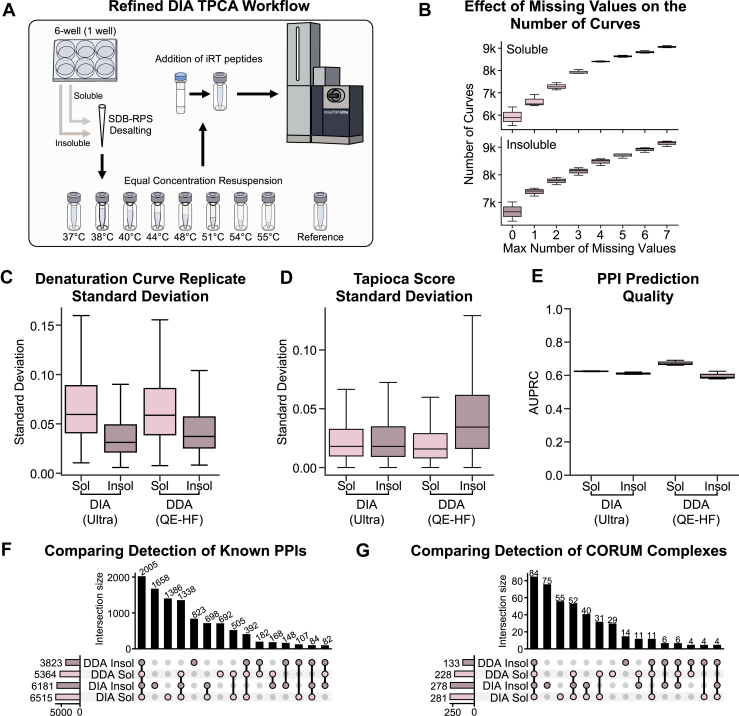
**Comparing optimized label-free DIA and TMT-DDA TPCA workflows.***A*, revised TPCA sample processing workflow for DIA analysis on timsTOF Ultra. *B*, box plot showing the number of curves by missing values for downstream PPI prediction at different thresholds of max number of missing values for any given curve. The line within the box represents the median value and the whiskers represent the ±1.5 interquartile range. *C*, box plot comparing the standard deviation of denaturation curves between replicates for all proteins for DIA and TMT-DDA soluble and insoluble experiments. Box plot elements are the same as in *B*. *D*, box plot comparing the standard deviation of Tapioca scores assigned to a given pair of proteins between replicates for all protein pairs for DIA and TMT-DDA soluble and insoluble experiments. Box plot elements are the same as in *B*. *E*, box plot comparing PPI prediction quality, measured by area under a precision recall curve (AUPRC), for DIA and TMT-DDA soluble and insoluble experiments. Box plot elements are the same as in *B*. *F*, upset plot comparing known PPIs, with experimental evidence from STRING (29), BIOGRID (27, 28), or REACTOME (45). *G*, upset plot comparing predicted assembled CORUM complexes (62). All experiments performed as N = 3 biological replicates.

To address this issue, we implemented several simple but effective modifications to the label-free DIA TPCA workflow to increase the number of denaturation curves with minimal missing values (Fig. 7*A* and Supplemental Table S7–S8). Firstly, we overlayed our conditional detection probability statistics with our previously published *in silico* temperature optimization analysis (39). This analysis led us to increase the number of temperature points to eight, which also afforded improved match-between-run (MBR) performance in DIA-NN analysis. Secondly, MBR performance was further improved with the addition of a reference sample, which was not subjected to thermal denaturation and was lysed in a more stringent buffer to enhance proteome coverage. Thirdly, we changed sample injection from equal volume to equal concentration, such that an equal amount of protein is injected on the instrument for each temperature point. Finally, we spiked iRT peptides into all samples to improve normalization and bypassed the use of a trap column to reduce loss of peptide identifications. These adjustments greatly improved the number of peptides and proteins detected across all temperatures and improved melting curve shape (Supplemental Figs. S15–S18, and S19, *A* and *B*). The number of proteins with no missing values in their denaturation curve also increased significantly, with ∼5900 and ∼6600 proteins in the soluble and insoluble fraction, respectively, having no missing values (Fig. 7*B* and Supplemental Fig. S19*C*). Conditional probabilities for detecting proteins at specific temperatures were similarly improved, particularly for the insoluble fraction (Supplemental Fig. S19*D*).

Using this refined workflow, we compared the label-free DIA TPCA (on a timsTOF Ultra) data to the TMT-DDA TPCA (on a Q-Exactive HF) data. We observed comparable reproducibility in both denaturation curves and Tapioca scores between the two workflows (Fig. 7, *C* and *D* and Supplemental Fig. S20*A*). Tapioca scores showed similar correlation between the TMT-DDA and label-free DIA TPCA workflows (Supplemental Fig. S20*B*). In terms of PPI prediction quality, the TMT-DDA TPCA soluble workflow outperformed its label-free DIA counterpart. However, the reverse was seen for the insoluble workflows, with the label-free DIA having better performance than the TMT-DDA TPCA workflow. Notably, when considering known PPIs, both the soluble and insoluble label-free DIA TPCA workflows outperformed their TMT-DDA TPCA counterparts (Fig. 7, *F* and *G*).

These refinements enabled the label-free DIA TPCA workflow to achieve performance levels comparable to, and in some areas superior to, the TMT-DDA TPCA workflow. It is worth noting that the TMT-DDA TPCA workflow has undergone extensive optimization to enhance PPI prediction quality (38, 39). Therefore, it is reasonable to anticipate that, as the optimization efforts will further continue, the label-free DIA TPCA workflow may surpass the TMT-DDA workflow in most aspects related to the reliable profiling of global PPI networks.

## Discussion

In this study, we explored how variations within the workflows of denaturation-based assays influence the measurement of global PPI networks. We produced paired TPCA and I-PISA datasets that include the measurement of insoluble fractions. While these workflows detected similar sets of proteins, they produced distinct denaturation profiles and PPI network predictions. By analyzing sequence-predicted protein physical properties, we found that both thermal and ion-based denaturation performed more reliably on larger and less structurally complex and intrinsically stable proteins. Ion-based denaturation was, up to a certain limit, able to denature proteins that were too hydrophobic and intrinsically stable for thermal denaturation.

Our results demonstrate that protein physical properties—and potentially subcellular localization—play critical roles in determining which PPIs are most likely to be detected by soluble and insoluble TPCA and I-PISA workflows. Using selected PPI networks (*i.e.*, cytoskeletal and mismatch repair) as case studies, we showed that these workflows captured different functional populations of a protein’s PPIs. Combining PPI predictions from multiple workflows allowed us to construct biologically informative and connected networks during both basal and perturbed (influenza A infection) states.

There are notable limitations to our analysis. We focused on amino acid sequence-predicted physical properties, which do not account for the influence of protein 3D structures on these properties or additional physical properties. Additionally, we considered only the average physical properties within PPIs, without accounting for changes induced by protein complex formation. Other types of interactions, such as protein-nucleic acid and protein-metabolite interactions, also affect denaturation profiles (34, 35, 131) and are likely to influence downstream PPI predictions. Furthermore, post-translational modifications on proteins may further alter protein denaturation susceptibility and dynamics across workflows, thereby influencing the subsets of PPIs observed. Despite these limitations, our findings begin to unravel previously unexplored determinants of PPI detection in denaturation-based assays and lay the groundwork for developing improved methodologies. For example, combining denaturation methods or exploring other denaturation approaches could reveal previously undetected populations of PPIs.

In addition to investigating how denaturation methods and the inclusion of insoluble fractions impact PPI detection, in this study, we examined the effects of reducing starting material and using a label-free DIA workflow. As expected, reducing the sample size decreased the number of identified peptides and proteins. However, PPI prediction quality remained consistent even with a ∼500× reduction in starting material. This result demonstrates the robustness of the TPCA workflow and its applicability to sample-limited contexts, such as rare cell types isolated from tissues, without compromising PPI prediction quality.

In agreement with previous reports (43, 44), our analyses show that missing values significantly limit the number of complete denaturation curves in label-free DIA workflows compared to TMT-DDA workflows. However, we demonstrate that simple modifications to the label-free DIA TPCA workflow enable near-equal performance to TMT-DDA TPCA in terms of the number and quality of complete curves. Further improvements, such as spiking in a reference sample (44), could enhance the label-free DIA workflow. Additionally, we identified specific temperature ranges that contribute most to the observed missing values in soluble and insoluble label-free DIA TPCA workflows, providing actionable targets for optimizing curve completeness.

Overall, this study highlights critical experimental considerations for improving how researchers probe global PPI networks using denaturation-based assays. Notably, it demonstrates the utility of insoluble fractions in these workflows, opening new avenues for enhancing assay performance and broadening their applicability.

## Data Availability

The mass spectrometry proteomics data reported in this paper have been deposited in the ProteomeXchange Consortium (132) via the PRIDE (133) partner repository with the dataset identifiers PXD058773 (10.6019/PXD058773) and PXD068620. Full peptide-level search results (containing sequence, m/z, modifications, scores, etc.) reports are available on Figshare (https://doi.org/10.6084/m9.figshare.30153100, https://doi.org/10.6084/m9.figshare.29089946). All Tapioca PPI predictions are available on Figshare (https://doi.org/10.6084/m9.figshare.30152950, https://doi.org/10.6084/m9.figshare.29089724, https://doi.org/10.6084/m9.figshare.29089289).

## Supplemental data

This article contains supplemental data.

## Conflict of interest

The authors declare that they do not have any conflicts of interest with the content of this article.
