# Supplementary material for: Exploring How Workflow Variations in Denaturation-Based Assays Impact Global Protein–Protein Interaction Predictions
Source: Mol Cell Proteomics. 2025 Dec 11;25(2):101479. doi: 10.1016/j.mcpro.2025.101479 (PMC12829148; doi:10.1016/j.mcpro.2025.101479)
Supplement: Supplementary Figures [file mmc9.pdf]

Supplementary Material for

Exploring How Workflow Variations in Denaturation-Based Assays Impact Global Protein-Protein Interaction Predictions

Tavis. J. Reed[1,2,3], Laura M. Haubold[3], Josiah E. Hutton[3], Olga G. Troyanskaya[1,2,4,*], Ileana M. Cristea[1, 3,*]

[1]Lewis-Sigler Institute for Integrative Genomics, Princeton University, Carl Icahn Laboratory, Princeton, NJ, 08544
[2]Department of Computer Science, Princeton University, 35 Olden Street, Princeton, NJ, 08540
[3]Department of Molecular Biology, Princeton University, 119 Lewis Thomas Laboratory, Princeton, NJ, 08544
[4]Flatiron Institute, Simons Foundation, New York City, NY 10001, USA

This Supplementary Information Contains:

Supplementary Figures S1 to S20
Legends for tables S1 to S8

Other Supplementary Material for this manuscript includes the following:

Tables S1 to S8

Diagram of Soluble and Insoluble Fractions of TPCA and I-PISA Experimental Workflows

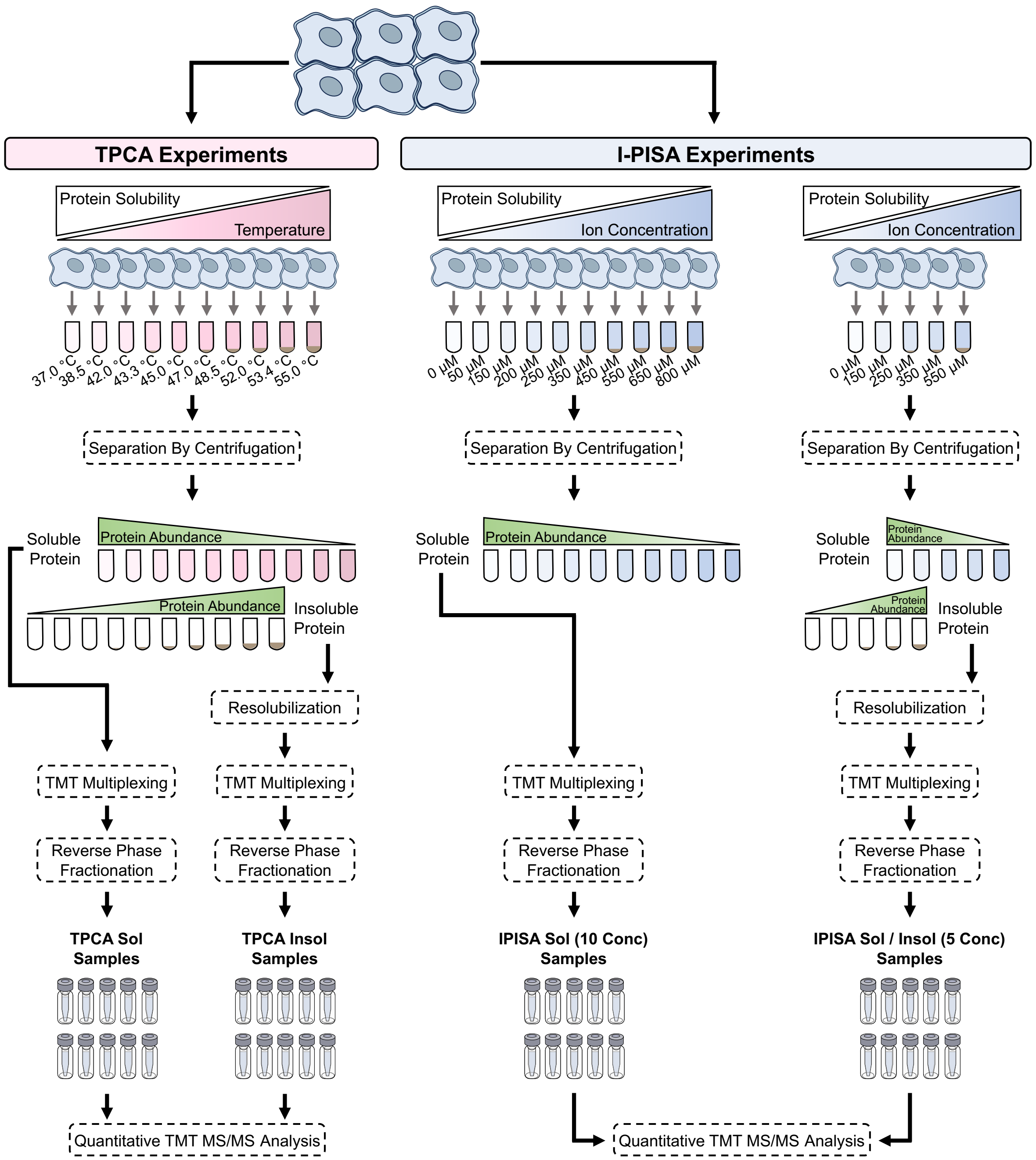

Fig. S1. Schematic overview of soluble and insoluble TPCA and I-PISA experiments. Skin epithelial (A375) cells were subjected to either TPCA or I-PISA workflows. For TPCA samples, ten temperature points were used for denaturation, and both the soluble and insoluble fractions were analyzed. For I-PISA samples, either ten or five concentration points were used for denaturation, and the soluble fractions of both and the insoluble fractions of the five-concentration samples were analyzed.

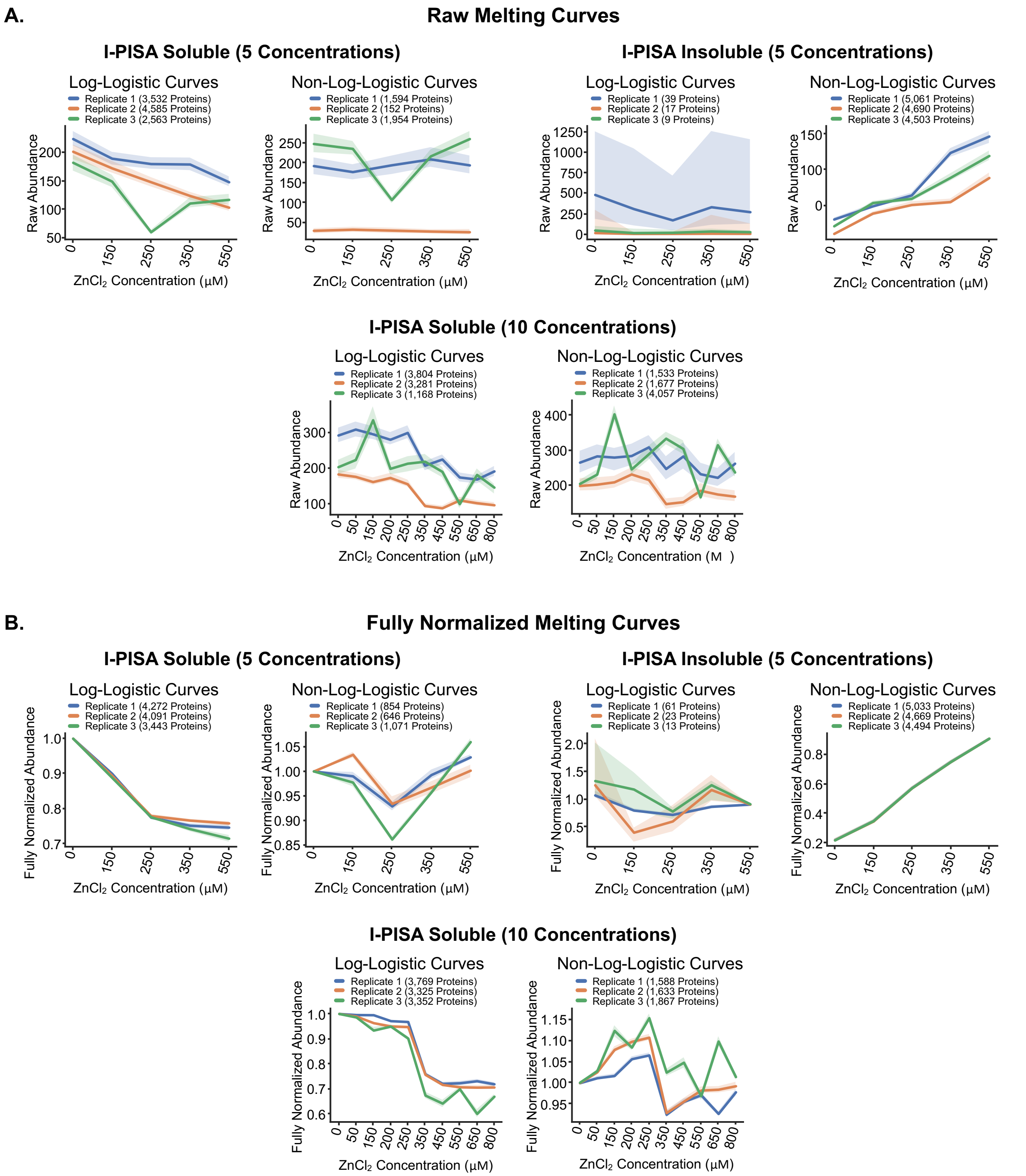

Fig. S2. I-PISA soluble and insoluble melting curve profiles. A, The I-PISA log-logistic and non-log logistic raw melting curves. **B,** The I-PISA log-logistic and non-log logistic normalized melting curves. For all plots, the solid line represents the median value and the shaded region represents the 95% confidence interval.

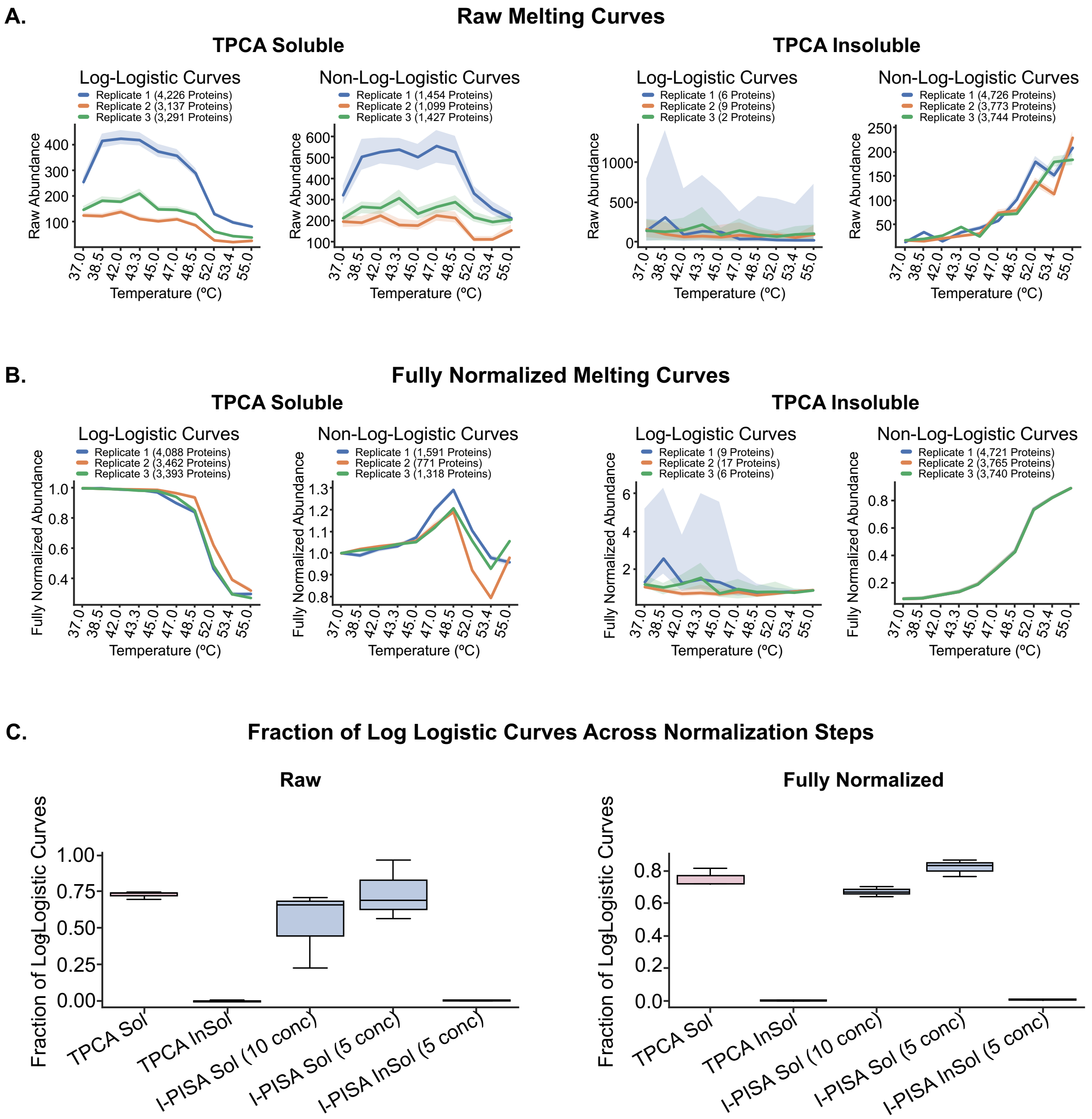

Fig. S3. TPCA soluble and insoluble melting curve profiles and fraction of log-logistic curves across workflows. A, The TPCA log-logistic and non-log logistic raw melting curves. **B,** The TPCA log-logistic and non-log logistic normalized melting curves. The solid line represents the median value and the shaded region represents the 95% confidence interval. **C,** The fraction of proteins with log-logistic melting/denaturation curves per soluble and insoluble TPCA and I-PSIA workflow. The line within the box represents the median value and the whiskers represent the ±1.5 interquartile range.

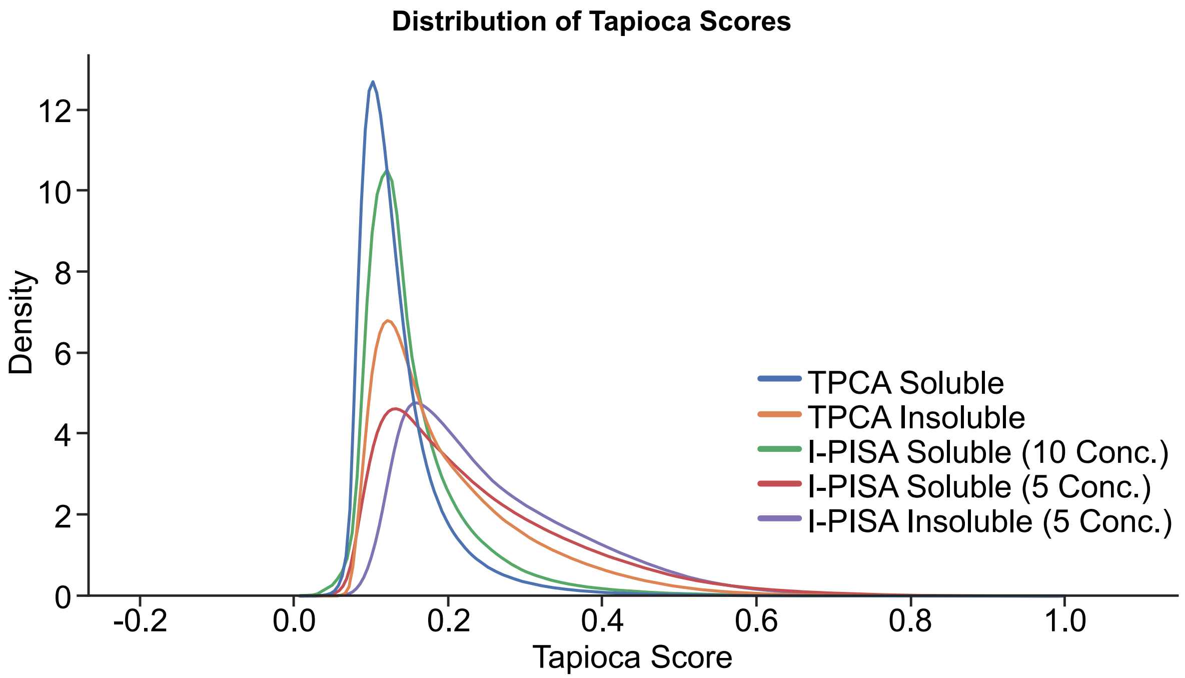

Fig. S4. Tapioca score distributions. The distributions of Tapioca scores for soluble and insoluble TPCA and I-PISA experiments.

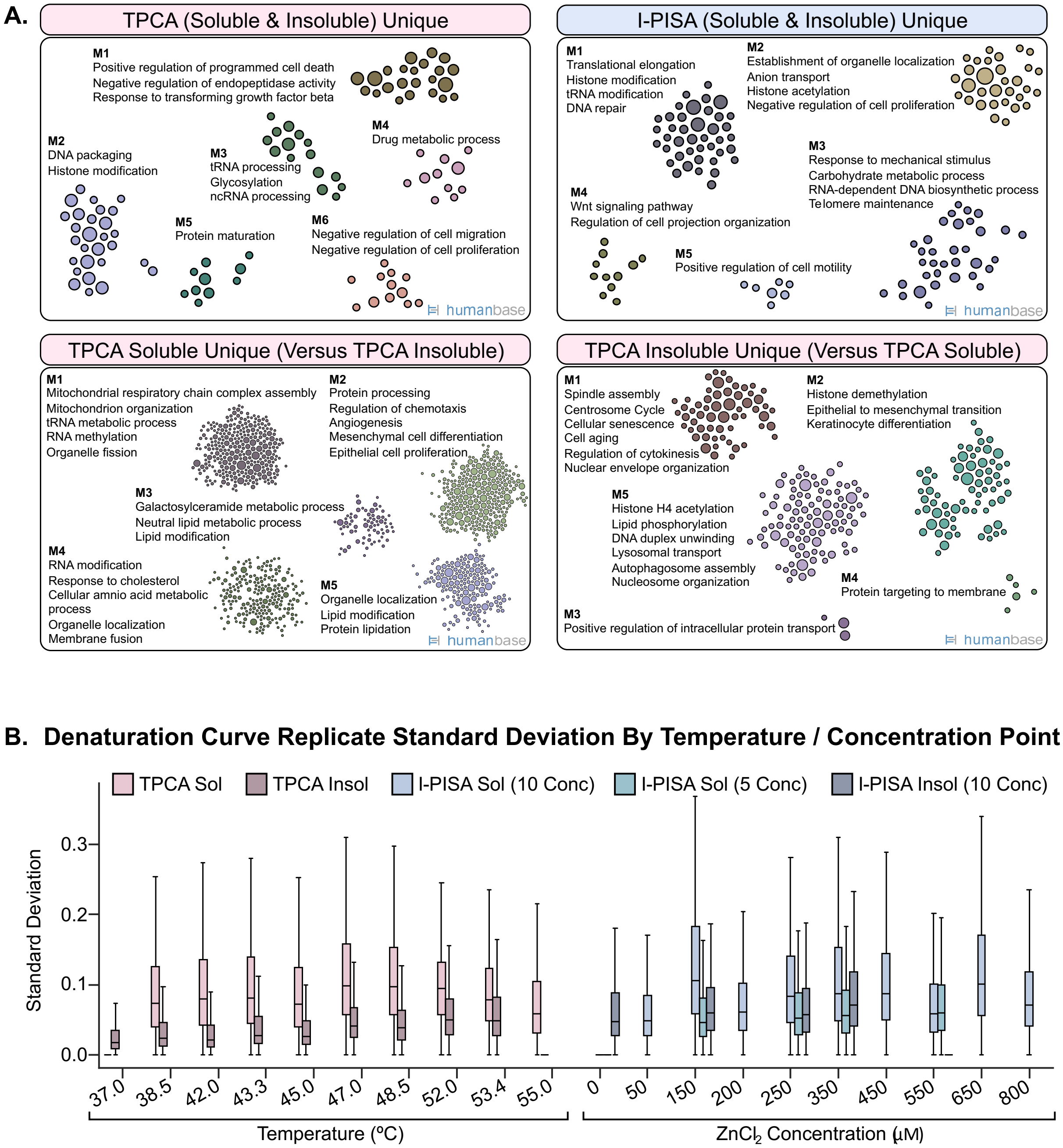

Fig. S5. GO Term enrichment of uniquely detected proteins and denaturation curve standard deviation. A, GO term enrichment of proteins uniquely detected when comparing different sets of experimental conditions. GO term enrichment was performed using HumanBase (https://hb.flatironinstitute.org/). M# (ex. M1) represents a functional module identified by HumanBase. In the plot, the nodes (circles/dots) represent proteins and are colored by the functional module that the given protein belongs to. The size of the node indicates the relative number of other proteins with which the given protein has a high degree of functional relatedness, with a larger node indicating a greater number of proteins. See supplementary table S2 for complete list of proteins and GO terms per module. **B,** The standard deviation of denaturation curves, per temperature or $ZnCl_2$ concentration, between replicates for all

proteins in a given experiment. The line within the box represents the median value and the whiskers represent the ±1.5 interquartile range.

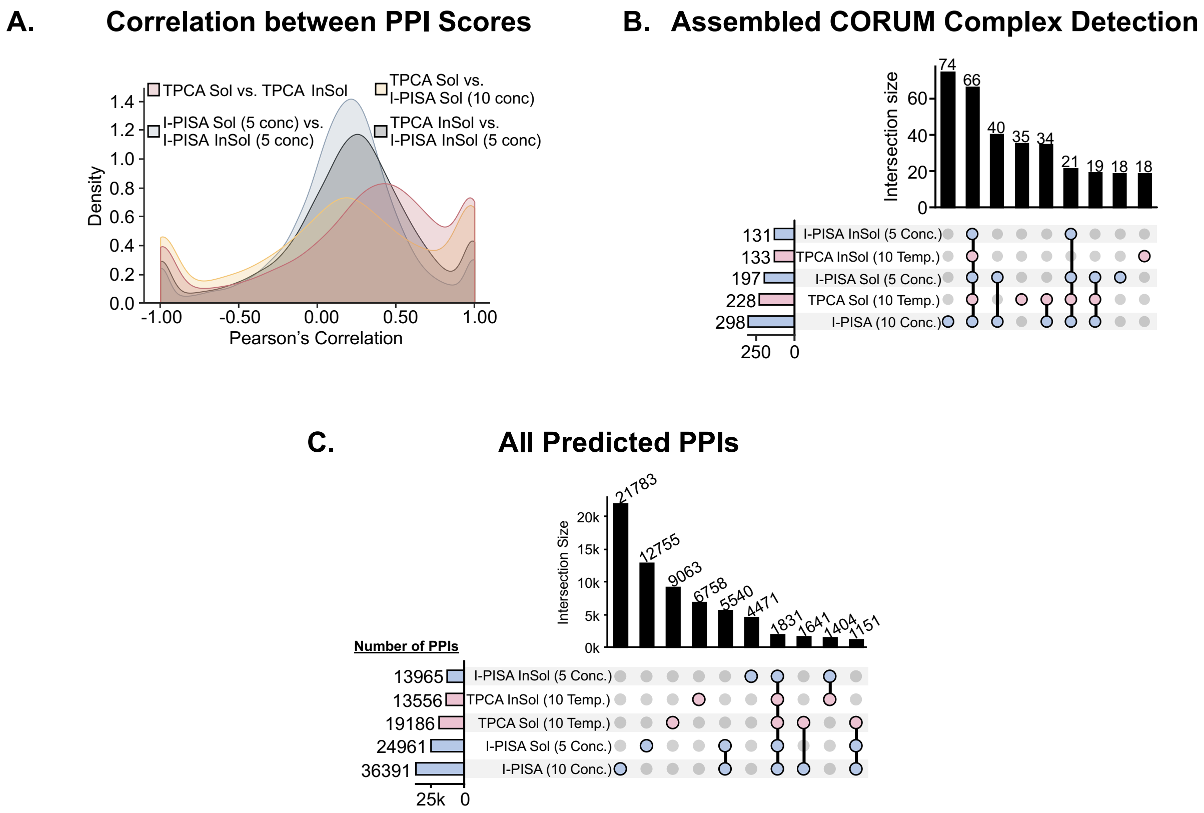

Fig S6. Comparison of detected PPIs. A, Distributions of the Pearson's correlation of PPI scores for a given protein for all proteins commonly detected between a given pair of experimental conditions. Only PPIs that achieved a score of greater than or equal to 0.5 in at least one of the experimental conditions being compared were considered during calculations. **B,** Upset plot comparing predicted assembled CORUM complexes across all soluble and insoluble TPCA and I-PISA experiments. **C,** Upset plot comparing all predicted PPIs across all soluble and insoluble TPCA and I-PISA experiments.

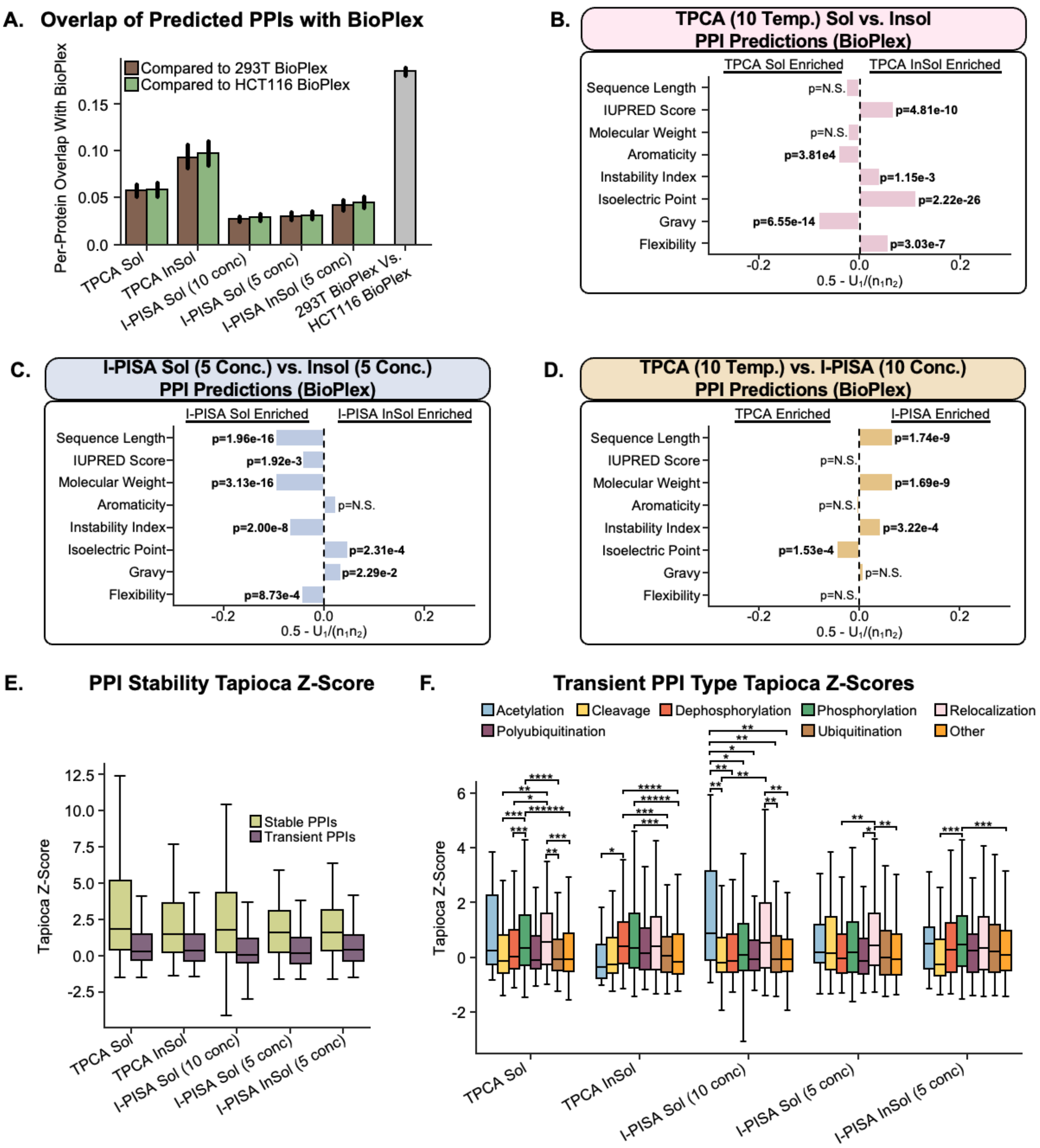

Fig S7. Comparing predicted PPIs to BioPlex and exploring stable versus transient PPI prediction. A, Bar plot showing the fraction overlap of PPIs commonly detected, per individual protein, between 293T (brown) or HCT116 (green) BioPlex datasets and soluble and insoluble TPCA and I-PISA workflows. The grey bar represents the fraction overlap of PPIs commonly detected, per individual protein, between 293T and HCT116 datasets. **B, C, D,** Bar plot showing the U-statistic derived values (X-axis) and associated multiple hypothesis test corrected P-value from the comparison of different sequence predicted physical properties of known PPIs predicted as assembled from different workflows (see Methods). **E,** Boxplot showing the distribution of Tapioca z-scores between stable and transient PPIs (see Methods) across

workflows The line within the box represents the median value and the whiskers represent the ±1.5 interquartile range. **F,** Boxplot showing the distribution of Tapioca z-scores for different types of transient PPIs (see Methods) across workflows. Only significant p-values between transient PPI types within a single workflow are represented. Boxplot elements are the same as in Fig. 3E. Across all panels of this figure all p-values were calculated using a two-sided Mann-Whitney U test followed by multiple hypothesis test correction using the Bonferroni correction method. For (*) representations of p-values, * = p-value ≤ 0.05, ** = p-value ≤ 0.001, *** = p-value ≤ 0.0001, **** = p-value ≤ 0.00001, ***** = p-value ≤ 0.000001, ****** = p-value ≤ 0.0000001.

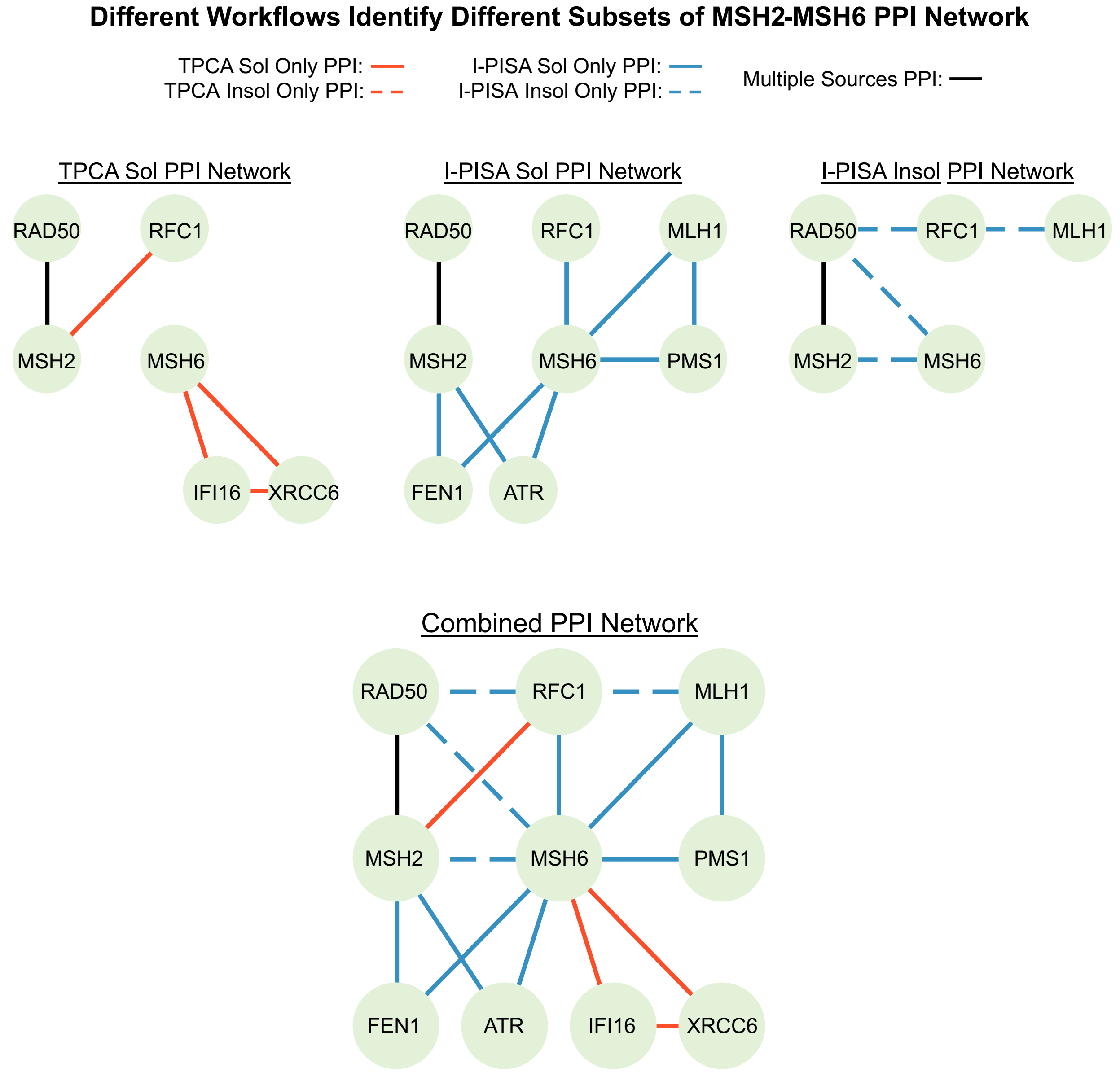

Fig S8. Comparison of the MSH2-MSH6 PPI network as detected by TPCA and I-PISA soluble and insoluble workflows. Example PPI network highlighting PPIs uniquely detected by different workflows.

Soluble and Insoluble Melting Curves of Regularly and Irregularly Behaved Proteins

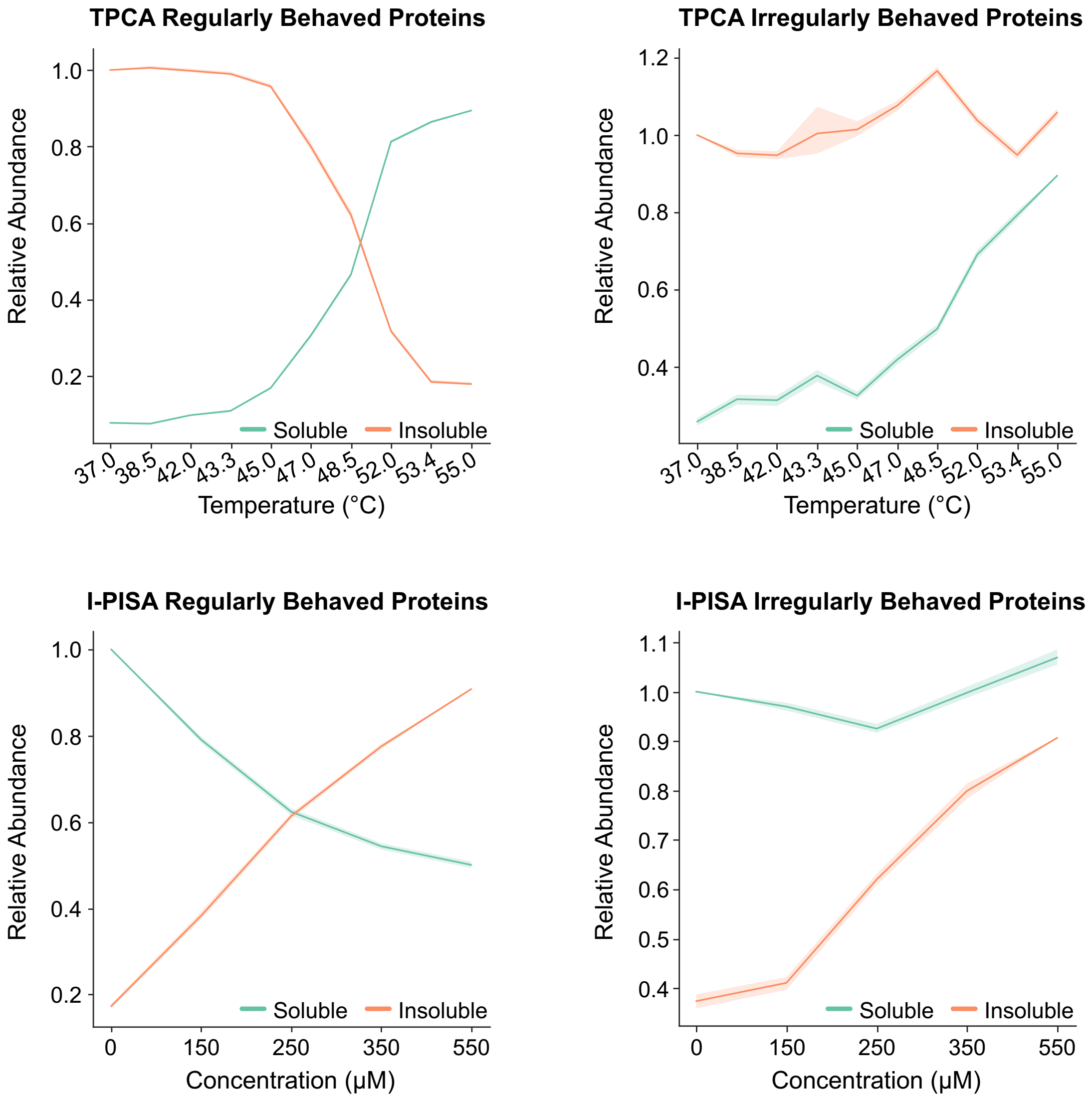

Fig S9. Average soluble and insoluble denaturation curve profiles of regularly and irregularly behaved proteins. The average soluble and insoluble melting profiles of proteins separated by regular and irregular behavior for both the TPCA and I-PISA workflows. The solid line represents the median value and the shaded region represents the 95% confidence interval.

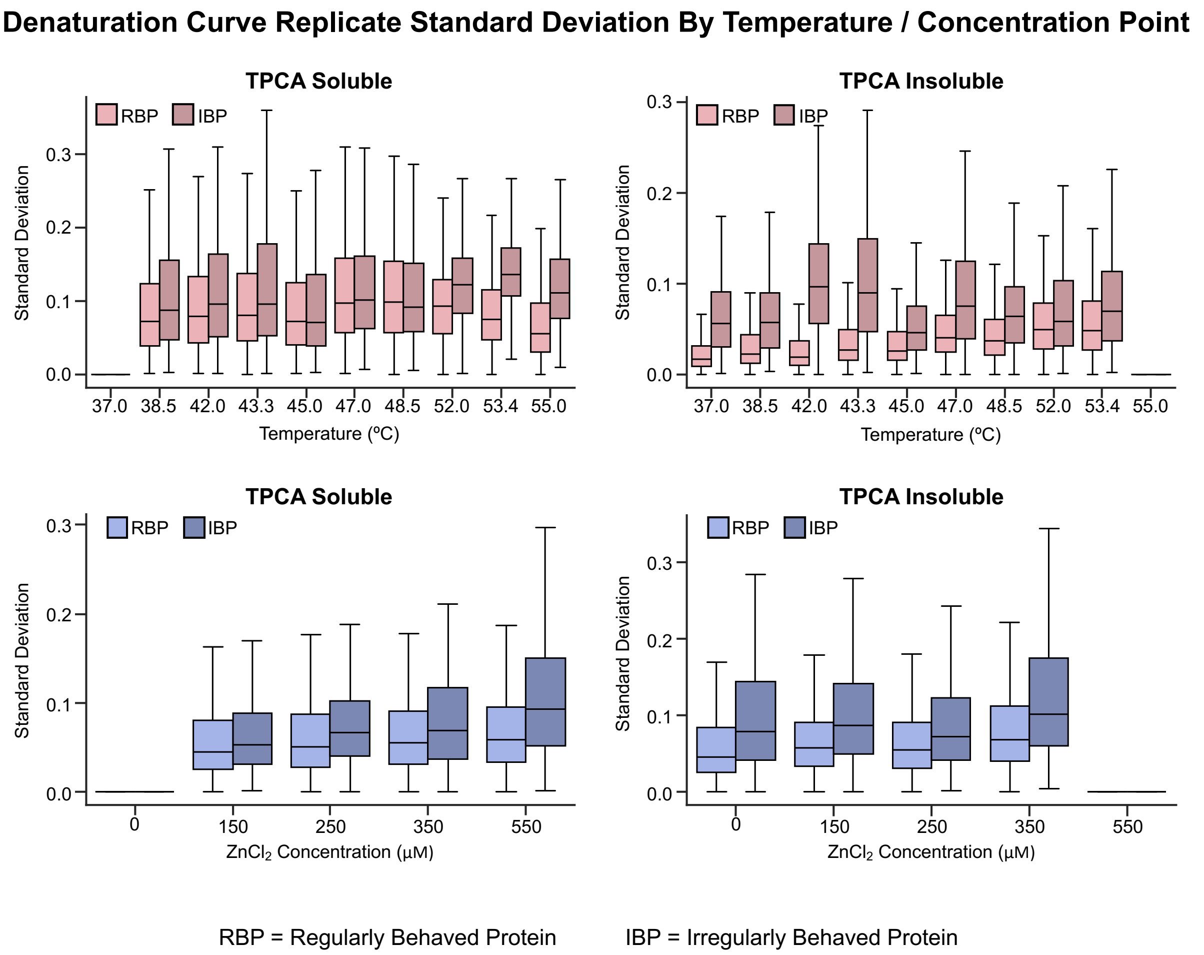

Fig. S10. TPCA and I-PISA denaturation curve standard deviation plots of regularly and irregularly behaved proteins. The standard deviation of denaturation curves, per temperature or $ZnCl_2$ concentration, between replicates for all proteins, split between regularly and irregularly behaved proteins, in a given experiment. The line within the box represents the median value and the whiskers represent the ±1.5 interquartile range.

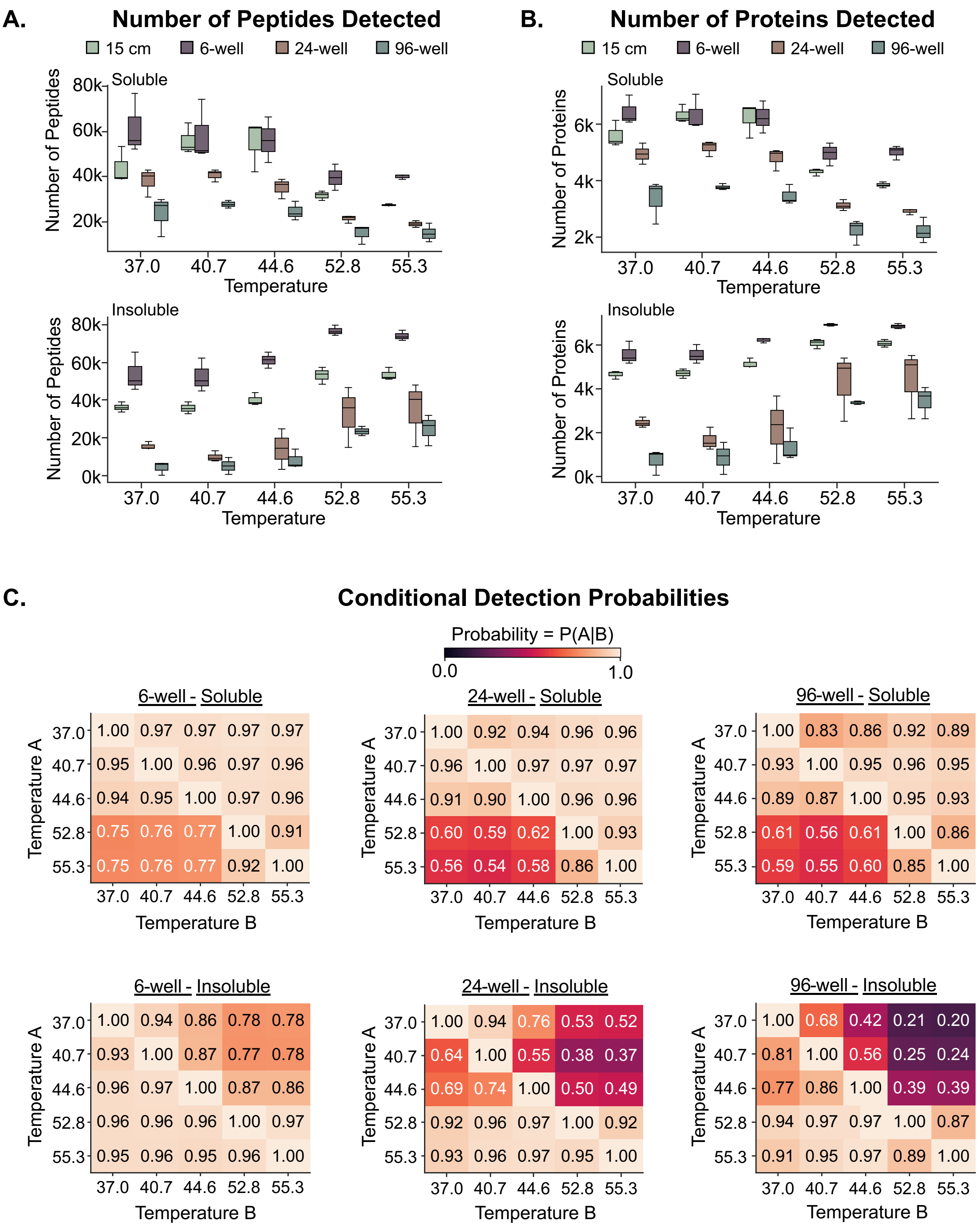

Fig S11. Additional assessment of DIA TPCA timsTOF Ultra data. A, Box plots showing the number of peptides detected per temperature per experimental condition. The line within the box represents the median value and the whiskers represent the ±1.5 interquartile range. **B,**

Box plots showing the number of proteins detected per temperature per experimental condition. Box plot elements are the same as in Fig S10A. **C,** The conditional probabilities of detecting a protein at temperature A given the protein was detected at temperature B in a given experimental condition.

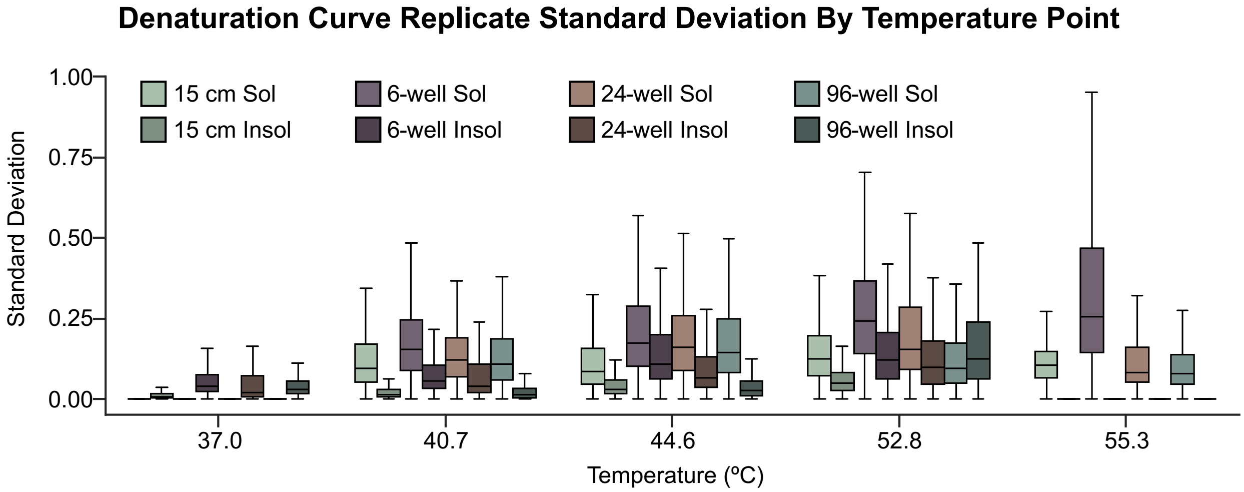

Fig. S12. TPCA and I-PISA denaturation curve standard deviation plots of regularly and irregularly behaved proteins. The standard deviation of denaturation curves, per temperature, between replicates for all proteins, for soluble and insoluble 15 cm, 6-well, 24-well, and 96-well experiments. The line within the box represents the median value and the whiskers represent the ±1.5 interquartile range.

A.

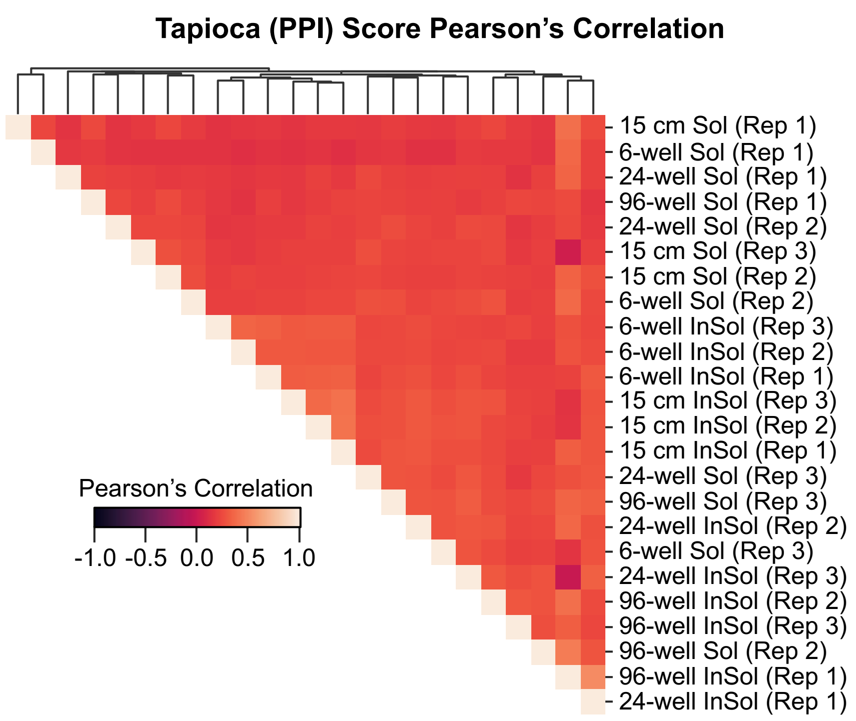

B.

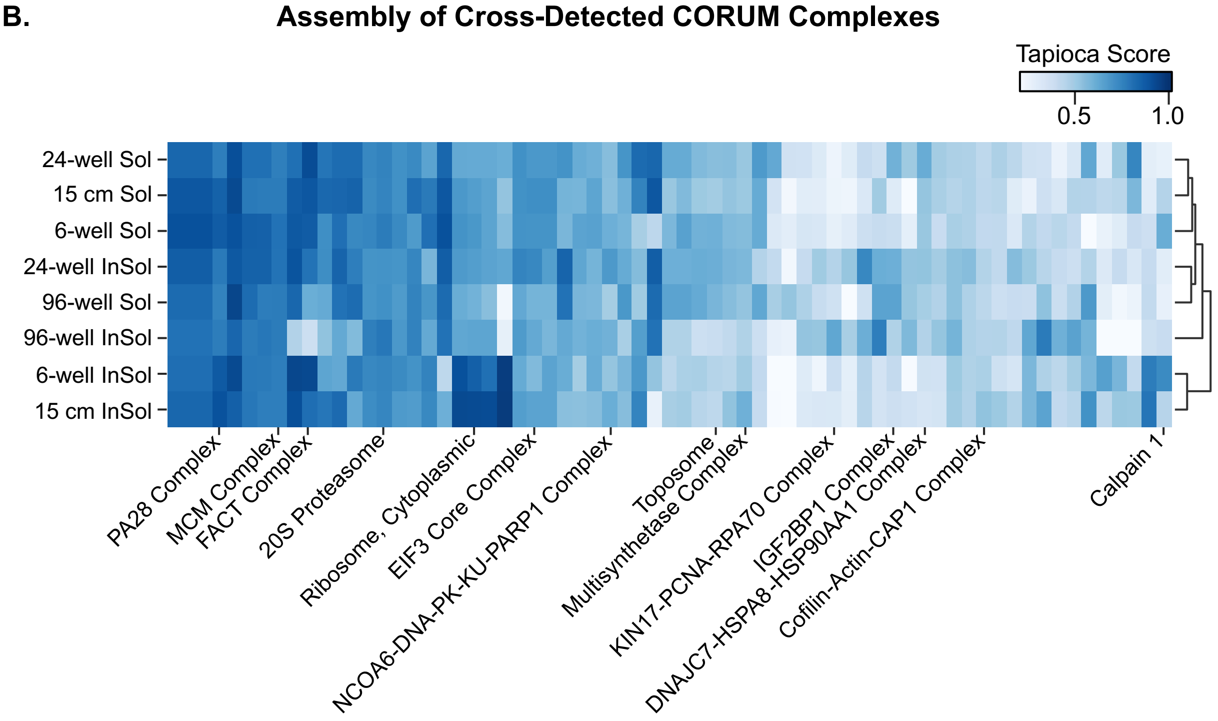

Fig S13. Comparing PPI scores and assembled CORUM complexes across DIA TPCA timsTOF Ultra data. A, Clustermap of the median Pearson's correlation of PPI scores between a given pair of experimental conditions. **B,** Clustermap of predicted CORUM complex assembly (by

Tapioca score) across experimental conditions. Each column represents a single CORUM complex.

A.

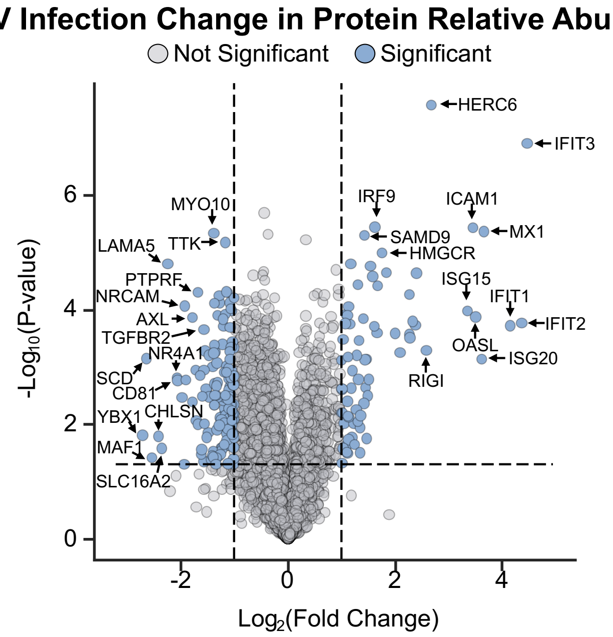
IAV Infection Change in Protein Relative Abundance
Not Significant
Significant
-Log10(P-value)
Log2(Fold Change)
HERC6
IFIT3
IRF9
ICAM1
MX1
SAMD9
HMGCR
ISG15
IFIT1
IFIT2
OASL
ISG20
RIGI
MYO10
TTK
LAMA5
PTPRF
NRCAM
AXL
TGFBR2
NR4A1
SCD
CD81
YBX1
CHLSN
MAF1
SLC16A2
0
2
4
6
-2
0
2
4

B.

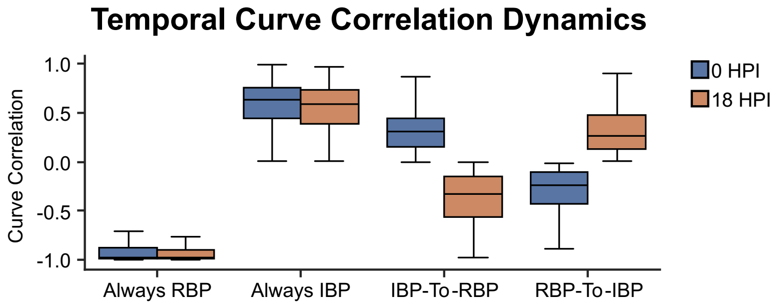
Temporal Curve Correlation Dynamics
Curve Correlation
1.0
0.5
0.0
-0.5
-1.0
0 HPI
18 HPI
Always RBP
Always IBP
IBP-To-RBP
RBP-To-IBP

C.

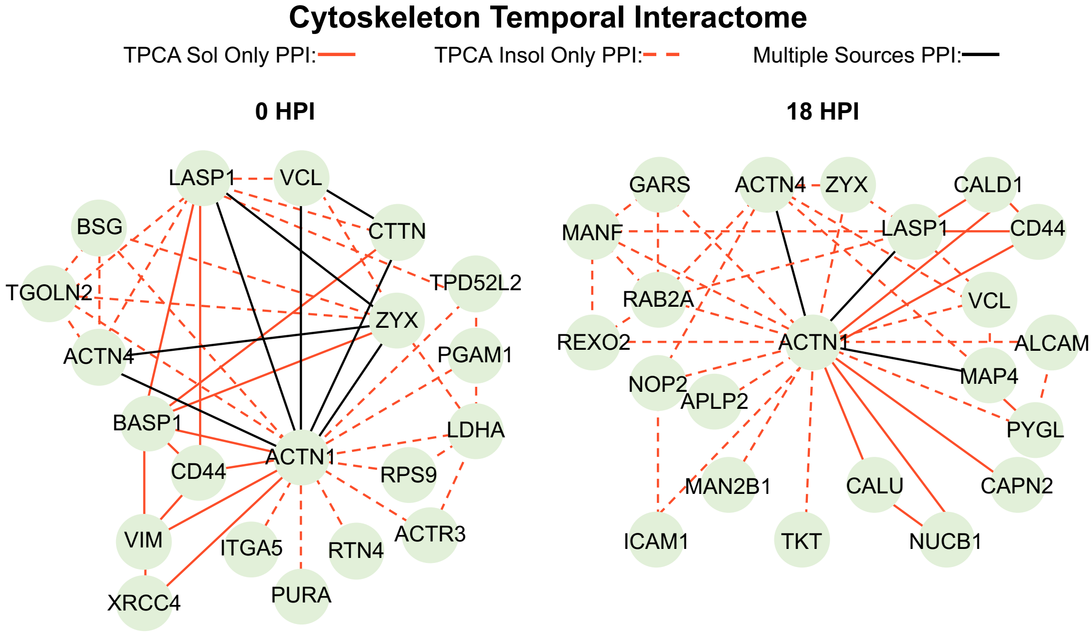
Cytoskeleton Temporal Interactome
TPCA Sol Only PPI:
TPCA Insol Only PPI:
Multiple Sources PPI:
0 HPI
18 HPI
LASP1
VCL
BSG
CTTN
TPD52L2
TGOLN2
ZYX
ACTN4
PGAM1
BASP1
LDHA
CD44
ACTN1
RPS9
VIM
ITGA5
RTN4
ACTR3
XRCC4
PURA
GARS
ACTN4
ZYX
CALD1
MANF
LASP1
CD44
RAB2A
VCL
REXO2
ACTN1
ALCAM
NOP2
MAP4
APLP2
PYGL
MAN2B1
CALU
CAPN2
ICAM1
TKT
NUCB1

Fig. S14. Influenza A (IAV) infection-induced alterations in protein abundances and interaction network. A, Volcano plot showing host protein abundance changes during IAV infection. The horizontal line shows the p-value cutoff, $-\log_{10}(0.05)$, and the vertical lines show the lower- and upper-fold change cutoffs, $\pm \log_2(2)$, used for calling a change significant. Significant proteins are colored blue and top up and down regulated proteins are labeled. The p-value was calculated using a two-sided student's T-test. **B,** The distributions of soluble and insoluble curve correlations per protein for proteins that are always RBPs (regularly behaved proteins), always IBPs (irregularly behaved proteins), or that transition between RBP and IBP classifications. The line within the box represents the median value and the whiskers represent the ±1.5 interquartile range. **C,** Temporal PPI networks centered on ACTN1. Edges between nodes represent the workflow which detected the given PPI, TPCA soluble, TPCA insoluble, or both.

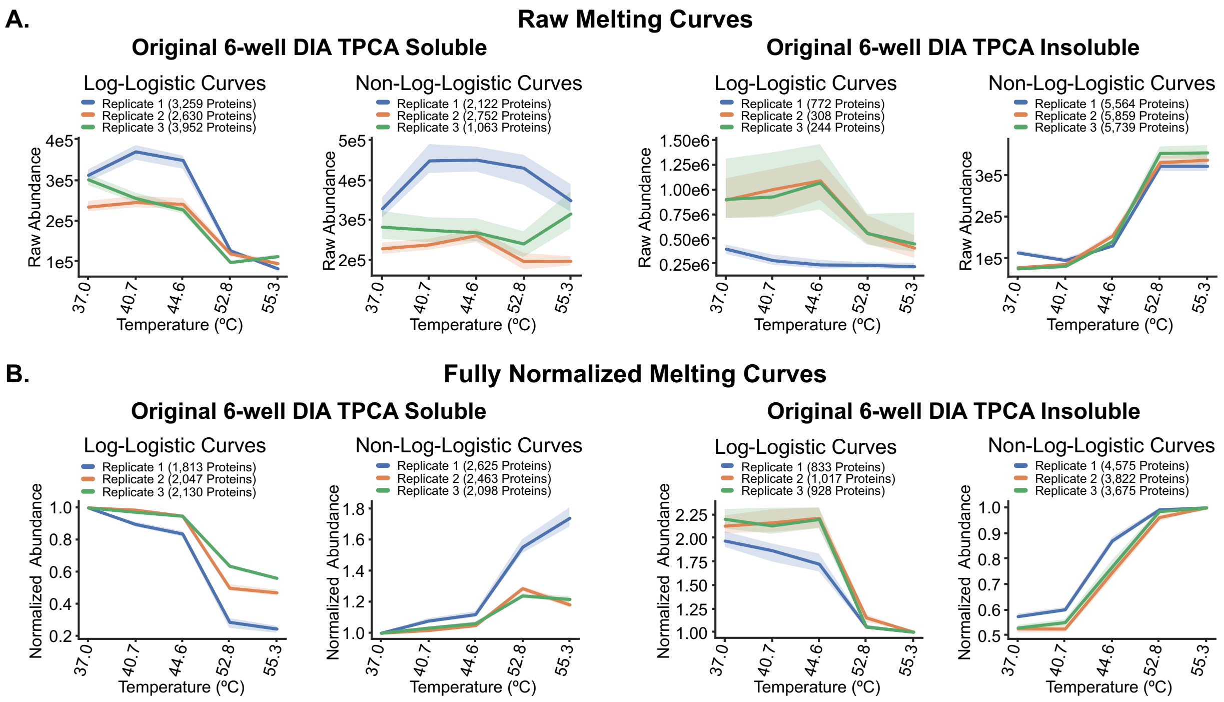

Fig. S15. Raw and fully normalized soluble and insoluble melting curve profiles for original 6-well DIA TPCA data. A, The raw soluble and insoluble log-logistic and non-log logistic melting curves for the original 6-well DIA TPCA data. **B,** The fully normalized soluble and insoluble log-logistic and non-log logistic melting curves for the original 6-well DIA TPCA data. For all plots, the solid line represents the median value, and the shaded region represents the 95% confidence interval.

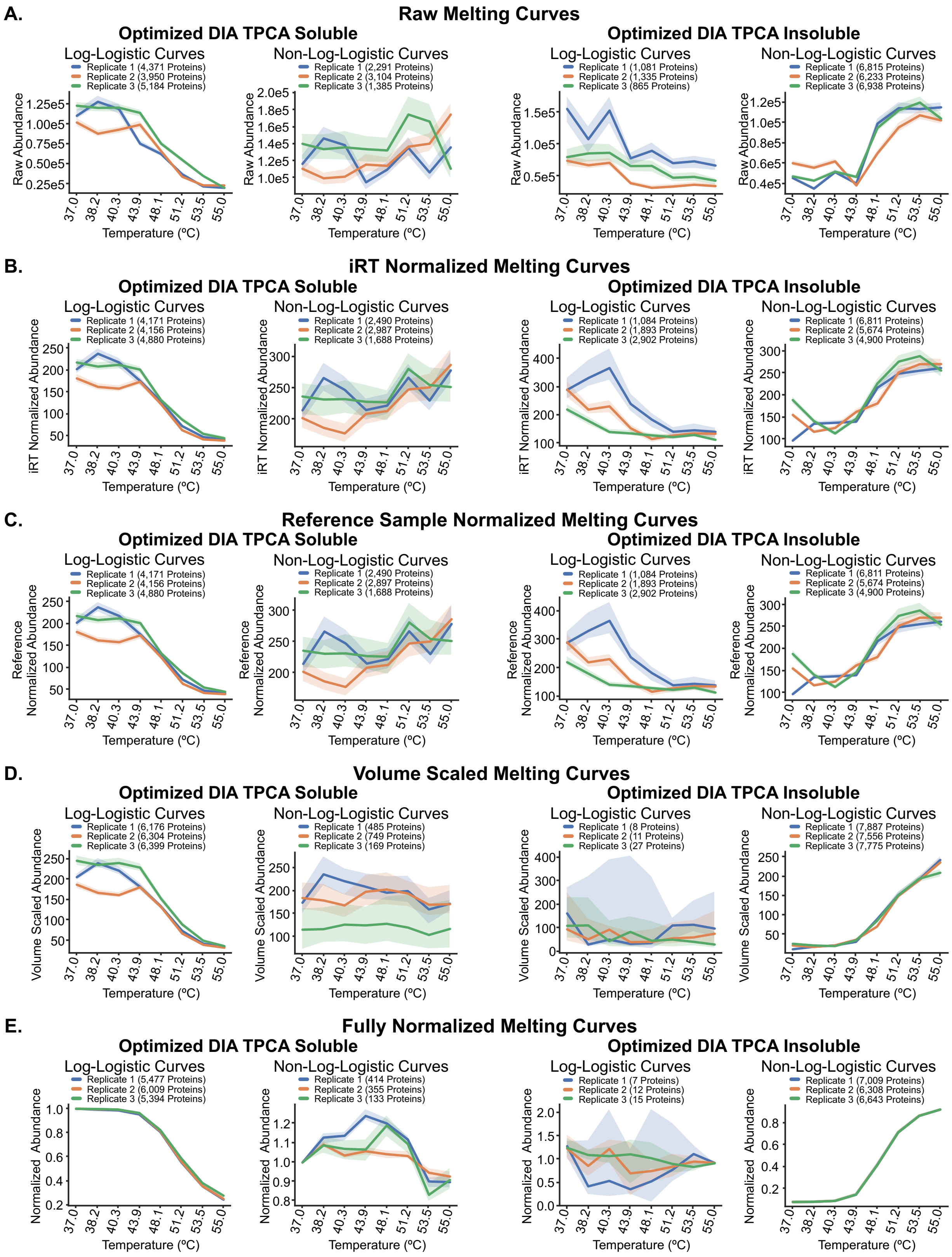
A.
Raw Melting Curves
Optimized DIA TPCA Soluble
Log-Logistic Curves
Replicate 1 (4,371 Proteins)
Replicate 2 (3,950 Proteins)
Replicate 3 (5,184 Proteins)
Non-Log-Logistic Curves
Replicate 1 (2,291 Proteins)
Replicate 2 (3,104 Proteins)
Replicate 3 (1,385 Proteins)
Optimized DIA TPCA Insoluble
Log-Logistic Curves
Replicate 1 (1,081 Proteins)
Replicate 2 (1,335 Proteins)
Replicate 3 (865 Proteins)
Non-Log-Logistic Curves
Replicate 1 (6,815 Proteins)
Replicate 2 (6,233 Proteins)
Replicate 3 (6,938 Proteins)
Raw Abundance
Temperature (ºC)
B.
iRT Normalized Melting Curves
Optimized DIA TPCA Soluble
Log-Logistic Curves
Replicate 1 (4,171 Proteins)
Replicate 2 (4,156 Proteins)
Replicate 3 (4,880 Proteins)
Non-Log-Logistic Curves
Replicate 1 (2,490 Proteins)
Replicate 2 (2,987 Proteins)
Replicate 3 (1,688 Proteins)
Optimized DIA TPCA Insoluble
Log-Logistic Curves
Replicate 1 (1,084 Proteins)
Replicate 2 (1,893 Proteins)
Replicate 3 (2,902 Proteins)
Non-Log-Logistic Curves
Replicate 1 (6,811 Proteins)
Replicate 2 (5,674 Proteins)
Replicate 3 (4,900 Proteins)
iRT Normalized Abundance
Temperature (ºC)
C.
Reference Sample Normalized Melting Curves
Optimized DIA TPCA Soluble
Log-Logistic Curves
Replicate 1 (4,171 Proteins)
Replicate 2 (4,156 Proteins)
Replicate 3 (4,880 Proteins)
Non-Log-Logistic Curves
Replicate 1 (2,490 Proteins)
Replicate 2 (2,897 Proteins)
Replicate 3 (1,688 Proteins)
Optimized DIA TPCA Insoluble
Log-Logistic Curves
Replicate 1 (1,084 Proteins)
Replicate 2 (1,893 Proteins)
Replicate 3 (2,902 Proteins)
Non-Log-Logistic Curves
Replicate 1 (6,811 Proteins)
Replicate 2 (5,674 Proteins)
Replicate 3 (4,900 Proteins)
Reference Normalized Abundance
Temperature (ºC)
D.
Volume Scaled Melting Curves
Optimized DIA TPCA Soluble
Log-Logistic Curves
Replicate 1 (6,176 Proteins)
Replicate 2 (6,304 Proteins)
Replicate 3 (6,399 Proteins)
Non-Log-Logistic Curves
Replicate 1 (485 Proteins)
Replicate 2 (749 Proteins)
Replicate 3 (169 Proteins)
Optimized DIA TPCA Insoluble
Log-Logistic Curves
Replicate 1 (8 Proteins)
Replicate 2 (11 Proteins)
Replicate 3 (27 Proteins)
Non-Log-Logistic Curves
Replicate 1 (7,887 Proteins)
Replicate 2 (7,556 Proteins)
Replicate 3 (7,775 Proteins)
Volume Scaled Abundance
Temperature (ºC)
E.
Fully Normalized Melting Curves
Optimized DIA TPCA Soluble
Log-Logistic Curves
Replicate 1 (5,477 Proteins)
Replicate 2 (6,009 Proteins)
Replicate 3 (5,394 Proteins)
Non-Log-Logistic Curves
Replicate 1 (414 Proteins)
Replicate 2 (355 Proteins)
Replicate 3 (133 Proteins)
Optimized DIA TPCA Insoluble
Log-Logistic Curves
Replicate 1 (7 Proteins)
Replicate 2 (12 Proteins)
Replicate 3 (15 Proteins)
Non-Log-Logistic Curves
Replicate 1 (7,009 Proteins)
Replicate 2 (6,308 Proteins)
Replicate 3 (6,643 Proteins)
Normalized Abundance
Temperature (ºC)

Fig. S16. Raw and normalized soluble and insoluble melting curve profiles for optimized 6-well DIA TPCA data. A, The raw soluble and insoluble log-logistic and non-log logistic melting curves for the optimized 6-well DIA TPCA data. **B,** The Indexed Retention Time (iRT) normalized soluble and insoluble log-logistic and non-log logistic melting curves for the optimized 6-well DIA TPCA data. **C,** The reference sample normalized soluble and insoluble log-logistic and non-log logistic melting curves for the optimized 6-well DIA TPCA data. **D,** The volume scaled soluble and insoluble log-logistic and non-log logistic melting curves for the optimized 6-well DIA TPCA data. **E,** The fully normalized soluble and insoluble log-logistic and non-log logistic melting curves for the optimized 6-well DIA TPCA. For all plots, the solid line represents the median value, and the shaded region represents the 95% confidence interval.

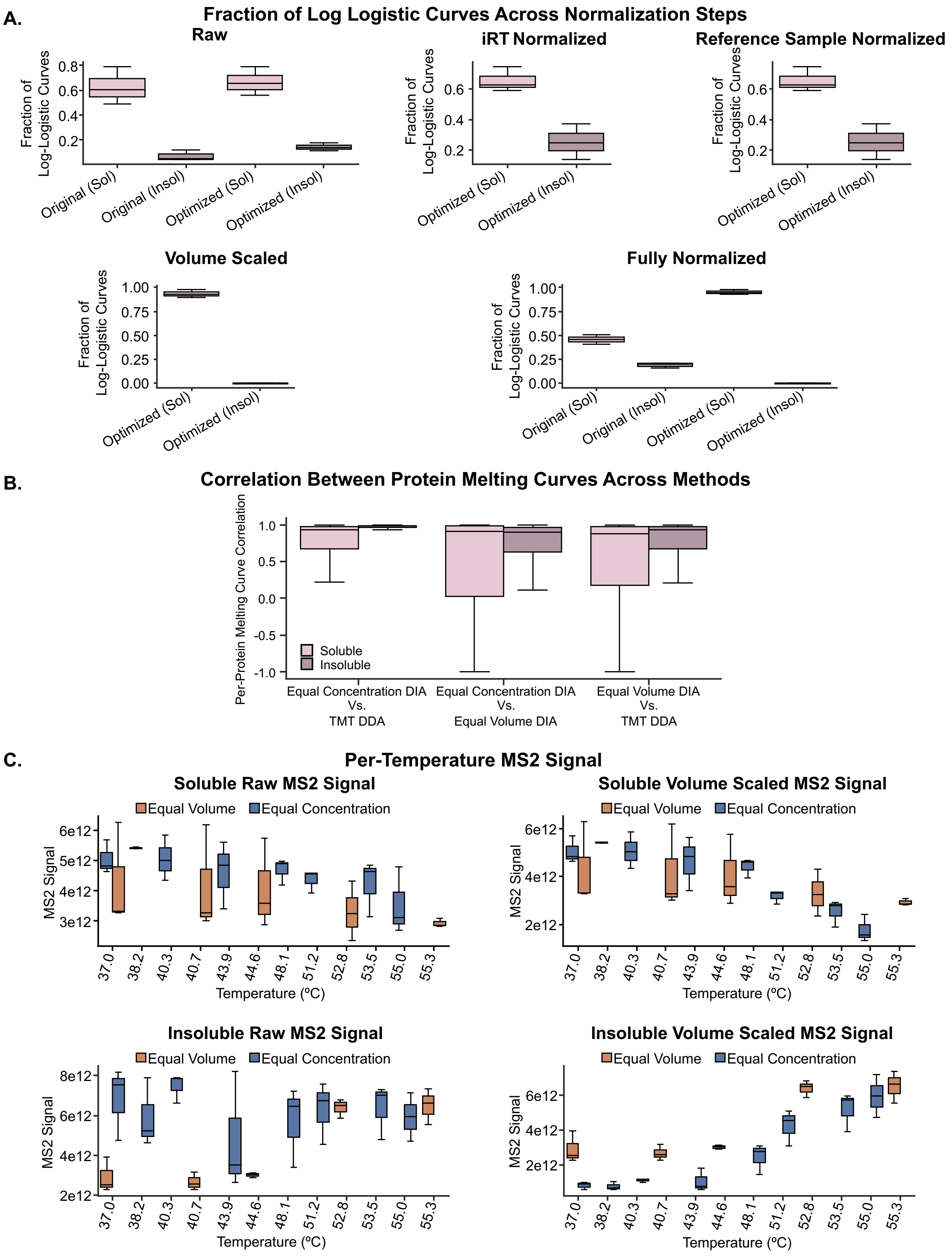

A.
Fraction of Log Logistic Curves Across Normalization Steps
Raw
iRT Normalized
Reference Sample Normalized
Fraction of Log-Logistic Curves
Original (Sol)
Original (Insol)
Optimized (Sol)
Optimized (Insol)
Volume Scaled
Fully Normalized
B.
Correlation Between Protein Melting Curves Across Methods
Per-Protein Melting Curve Correlation
Soluble
Insoluble
Equal Concentration DIA Vs. TMT DDA
Equal Concentration DIA Vs. Equal Volume DIA
Equal Volume DIA Vs. TMT DDA
C.
Per-Temperature MS2 Signal
Soluble Raw MS2 Signal
Soluble Volume Scaled MS2 Signal
Insoluble Raw MS2 Signal
Insoluble Volume Scaled MS2 Signal
Equal Volume
Equal Concentration
MS2 Signal
Temperature (ºC)

Fig. S17. Fraction of log-logistic curves and melting curve correlation across data acquisition methods and per-temperature MS2 signal before and after volume scaling. A, The fraction of proteins with log-logistic melting/denaturation curves for original and optimized 6-well DIA TPCA data per normalization step. **B,** The Pearson's correlation between fully normalized melting curves, per-protein, between equal volume (original 6-well DIA TPCA), equal concentration (optimized 6-well DIA TPCA) and TMT DDA TPCA. **B,** Boxplots showing the per temperature distribution of MS2 signal obtained from the .stats file output from DIA-NN. Plots contain data for equal volume, data from the 6-well 5 temperature DIA experiment, and equal concentration, data from the optimized 6-well 8 temperature DIA experiment. Plots are shown for soluble and insoluble fractions before and after scaling the values for equal concentration to account for the differential volumes of resuspension buffer used.

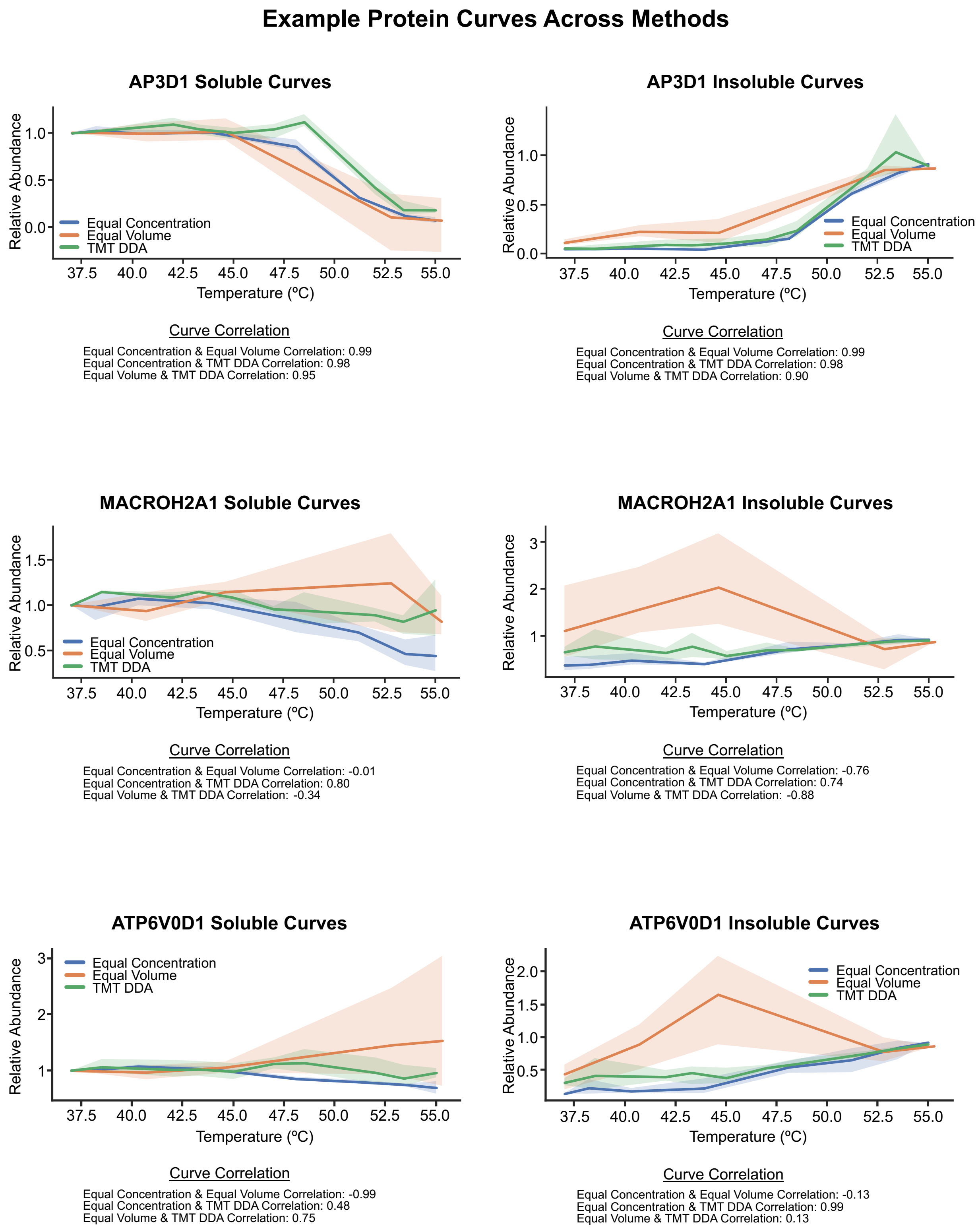

Fig. S18. Soluble and insoluble melting curves across workflows for selected proteins. Equal volume (original 6-well DIA TPCA workflow), equal concentration (optimized 6-well DIA TPCA workflow), and TMT DDA soluble and insoluble melting profiles for example proteins. For all

plots, the solid line represents the median value, and the shaded region represents the 95% confidence interval.

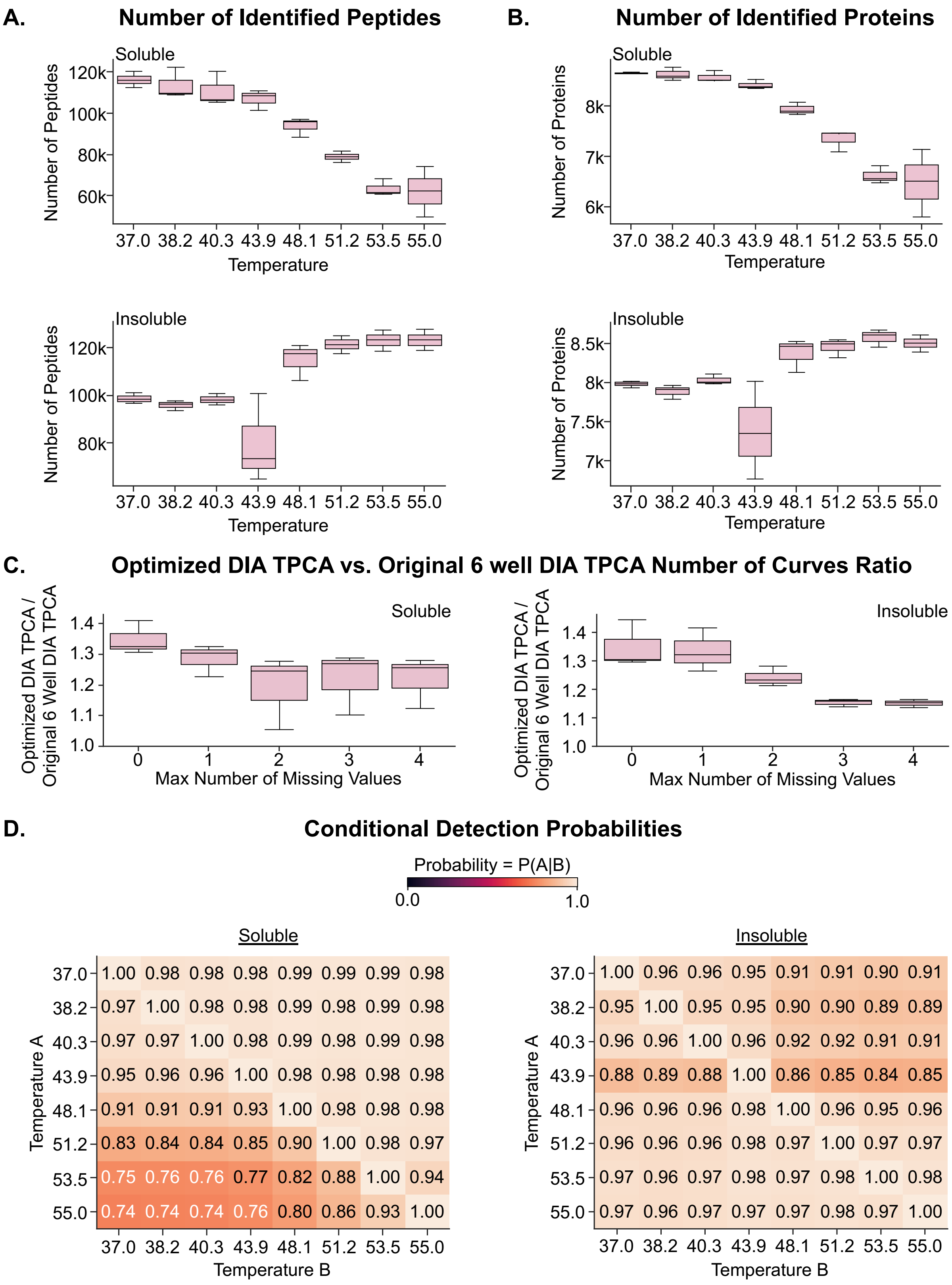
A.
Number of Identified Peptides
Soluble
Number of Peptides
120k
100k
80k
60k
37.0 38.2 40.3 43.9 48.1 51.2 53.5 55.0
Temperature
Insoluble
Number of Peptides
120k
100k
80k
37.0 38.2 40.3 43.9 48.1 51.2 53.5 55.0
Temperature
B.
Number of Identified Proteins
Soluble
Number of Proteins
8k
7k
6k
37.0 38.2 40.3 43.9 48.1 51.2 53.5 55.0
Temperature
Insoluble
Number of Proteins
8.5k
8k
7.5k
7k
37.0 38.2 40.3 43.9 48.1 51.2 53.5 55.0
Temperature
C.
Optimized DIA TPCA vs. Original 6 well DIA TPCA Number of Curves Ratio
Soluble
Optimized DIA TPCA / Original 6 Well DIA TPCA
1.4
1.3
1.2
1.1
1.0
0 1 2 3 4
Max Number of Missing Values
Insoluble
Optimized DIA TPCA / Original 6 Well DIA TPCA
1.4
1.3
1.2
1.1
1.0
0 1 2 3 4
Max Number of Missing Values
D.
Conditional Detection Probabilities
Probability = P(A|B)
0.0
1.0
Soluble
Temperature A
37.0 1.00 0.98 0.98 0.98 0.99 0.99 0.99 0.98
38.2 0.97 1.00 0.98 0.98 0.99 0.98 0.99 0.98
40.3 0.97 0.97 1.00 0.98 0.99 0.98 0.99 0.98
43.9 0.95 0.96 0.96 1.00 0.98 0.98 0.98 0.98
48.1 0.91 0.91 0.91 0.93 1.00 0.98 0.98 0.98
51.2 0.83 0.84 0.84 0.85 0.90 1.00 0.98 0.97
53.5 0.75 0.76 0.76 0.77 0.82 0.88 1.00 0.94
55.0 0.74 0.74 0.74 0.76 0.80 0.86 0.93 1.00
37.0 38.2 40.3 43.9 48.1 51.2 53.5 55.0
Temperature B
Insoluble
Temperature A
37.0 1.00 0.96 0.96 0.95 0.91 0.91 0.90 0.91
38.2 0.95 1.00 0.95 0.95 0.90 0.90 0.89 0.89
40.3 0.96 0.96 1.00 0.96 0.92 0.92 0.91 0.91
43.9 0.88 0.89 0.88 1.00 0.86 0.85 0.84 0.85
48.1 0.96 0.96 0.96 0.98 1.00 0.96 0.95 0.96
51.2 0.96 0.96 0.96 0.98 0.97 1.00 0.97 0.97
53.5 0.97 0.96 0.97 0.98 0.97 0.98 1.00 0.98
55.0 0.97 0.96 0.97 0.97 0.97 0.98 0.97 1.00
37.0 38.2 40.3 43.9 48.1 51.2 53.5 55.0
Temperature B

Fig S19. Additional assessment of DIA TPCA timsTOF Ultra data from the optimized workflow. A, Box plots showing the number of peptides detected per temperature per experimental condition. The line within the box represents the median value and the whiskers represent the ±1.5 interquartile range. **B,** Box plots showing the number of proteins detected per temperature per experimental condition. Box plot elements are the same as in Fig S18A. **C,** Box plots showing the ratio of number of proteins with set amounts of maximum missing values between the optimized DIA TPCA workflow and the original 6-well data. Box plot elements are the same as in Fig S18A. **D,** The conditional probabilities of detecting a protein at temperature A given the protein was detected at temperature B in a given experimental condition.

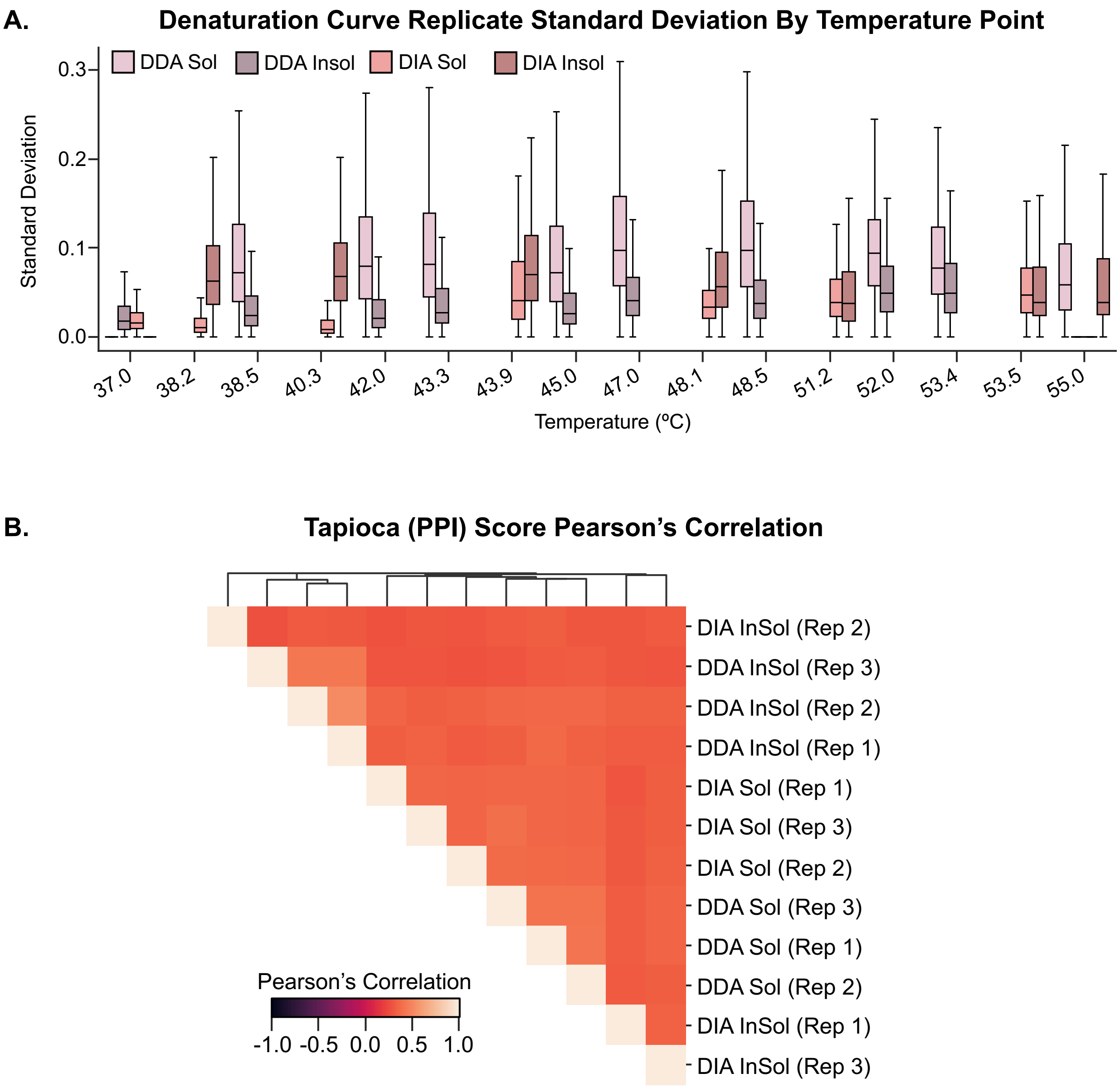

Fig. S20. Optimized DIA TPCA and TMT DDA TPCA curve standard deviation plots and comparing PPI scores across DIA TPCA timsTOF Ultra data from revised workflow and TMT DDA data. A, The standard deviation of denaturation curves, per temperature, between replicates for all proteins, for soluble and insoluble optimized DIA TPCA and TMT DDA TPCA experiments. The line within the box represents the median value and the whiskers represent the ±1.5 interquartile range. **B,** Clustermap of the median Pearson's correlation of PPI scores between a given pair of experimental conditions.

Table S1. (Separate file)
Spreadsheet containing raw and normalized data for TMT-DDA TPCA and I-PISA soluble and insoluble experiments.

Table S2. (Separate file)
HumanBase GO term enrichment of proteins; full list of modules and GO terms.

Table S3. (Separate file)
Spreadsheet containing raw and normalized data for label-free DIA TPCA 15 cm, 6-well, 24-well, and 96-well soluble and insoluble experiments.

Table S4 (Separate file)
Spreadsheet containing raw and normalized data for soluble and insoluble influenza A infection experiments.

Table S5. (Separate file)
Spreadsheet of fold change and p-values for Fig S13A volcano plot.

Table S6. (Separate file)
Spreadsheet of CORUM complex names and Tapioca scores for Fig 7B.

Table S7. (Separate file)
Spreadsheet containing raw and normalized data for the optimized label-free DIA TPCA 8 temperature soluble and insoluble experiments.

Table S8. (Separate file)
Optimized DIA-PASEF windows used for all DIA experiments. The table contains the ion mobility and m/z ranges for each for each isolation window, as well as which group each window belongs to, used during DIA MS2 scans.
